# Pyro-Phototronic Effect for Advanced Photodetectors and Novel Light Energy Harvesting

**DOI:** 10.3390/nano13081336

**Published:** 2023-04-11

**Authors:** Fangpei Li, Wenbo Peng, Yitong Wang, Mingyan Xue, Yongning He

**Affiliations:** 1 State Key Laboratory of Solidification Processing, Key Laboratory of Radiation Detection Materials and Devices, School of Materials Science and Engineering, Northwestern Polytechnical University, Xi’an 710072, China; 2 School of Microelectronics, Xi’an Jiaotong University, Xi’an 710049, China; 3 The Key Laboratory of Micro-Nano Electronics and System Integration of Xi’an City, Xi’an 710049, China

**Keywords:** pyro-phototronic effect, polarization charge, photodetector, light energy harvesting

## Abstract

Pyroelectricity was discovered long ago and utilized to convert thermal energy that is tiny and usually wasted in daily life into useful electrical energy. The combination of pyroelectricity and optoelectronic yields a novel research field named as *Pyro-Phototronic*, where light-induced temperature variation of the pyroelectric material produces pyroelectric polarization charges at the interfaces of semiconductor optoelectronic devices, capable of modulating the device performances. In recent years, the pyro-phototronic effect has been vastly adopted and presents huge potential applications in functional optoelectronic devices. Here, we first introduce the basic concept and working mechanism of the pyro-phototronic effect and next summarize the recent progress of the pyro-phototronic effect in advanced photodetectors and light energy harvesting based on diverse materials with different dimensions. The coupling between the pyro-phototronic effect and the piezo-phototronic effect has also been reviewed. This review provides a comprehensive and conceptual summary of the pyro-phototronic effect and perspectives for pyro-phototronic-effect-based potential applications.

## 1. Introduction

Energy generation and consumption is always one of the hottest topics in the energy-related research field. Beyond the traditional energy production utilizing coal and oil, many green energy sources have been explored and developed into practical applications, for instance, sunlight, wind, water, and heat. Solar cells are well established to efficiently harvest sunlight energy and convert it into electric energy [1,2,3,4]. For wind and water utilization, generally, their energy is first converted to mechanical energy and then to electric energy [5,6,7,8]. Prof. Zhong Lin Wang invented the first piezoelectric nanogenerator in 2007, adopting the ZnO nanowires to harvest tiny mechanical energy originating from the atomic force microscopy tip and produce a voltage output of about a few millivolts [9]. After that, the piezoelectric nanogenerators attracted great attention from worldwide research institutes and people, who became devoted to improving the energy conversion efficiency as well as the mechanical energy sources that could be harvested. Then in 2012, Prof. Zhong Lin Wang further invented the first triboelectric nanogenerator based on the combination between contact electrification and electrostatic induction [10], which is now proven to be originated from Maxwell’s displacement current [11,12,13]. From that time to now, the research fields and applications of nanogenerators have vastly expanded and developed quickly. Despite the abundant mechanical energy sources in human being’s daily life, heat energy sources can also be seen and found everywhere and every day, for example, the human body’s thermal energy, temperature variation in the surrounding environment, and so on. Consequently, the thermoelectric- and pyroelectric-effect-based nanogenerators are explored to harvest heat energy. Thermoelectric nanogenerator usually needs a relatively stable temperature difference to drive the motion of carriers in thermoelectric materials [14,15]. While pyroelectric nanogenerator mainly focuses on the transient temperature variation of pyroelectric materials [16,17,18]. More specifically speaking, once the temperature of a pyroelectric material is varied, then the corresponding amount of pyroelectric polarization charges would be induced in the pyroelectric material due to the pyroelectric effect, driving the carriers moving along the outside circuit to produce electricity. As a result, a pyroelectric nanogenerator should be more suitable to be utilized to harvest the unstable thermal energy sources in human being’s daily life, compared to a thermoelectric nanogenerator.

Beyond the general application of harvesting thermal energy, the pyroelectric effect has been further explored to cooperate with the photoelectric effect in semiconductor materials and devices, yielding a novel research field named *Pyro-phototronic*. The so-called pyro-phototronic effect was first coined by Prof. Zhong Lin Wang in 2015 [19]. In the first research exploring the pyro-phototronic effect, a perovskite/ZnO nanowires heterojunction was formed and operated in the self-powered mode, i.e., the applied voltage bias is 0 V. When the perovskite/ZnO nanowires heterojunction was illuminated by periodic UV laser, some interesting phenomena were observed; one rapid and sharp photocurrent peak occurs at the time UV laser turns on, and another corresponding rapid and sharp photocurrent peak with reverse direction occurs at the time UV laser turns off. After a series of careful experiments and discussion, these interesting phenomena are confirmed to originate from the light-induced transient temperature variation of the ZnO nanowires. Since ZnO not only is a piezoelectric material but also owns the pyroelectric property, this light-induced transient temperature variation of ZnO nanowires generates a large number of pyroelectric polarization charges at the perovskite/ZnO nanowires interface, thereby modulating the UV response performances. The results found that the response time was improved from a few seconds to dozens of microseconds, demonstrating an enhancement of about five orders of magnitude. In addition, the responsivity and detectivity were also both enhanced by over 300%. More importantly, the proposed pyro-phototronic-effect-enhanced perovskite/ZnO nanowires heterojunction photodetector operates at a zero-voltage bias, yielding not only a very low dark leakage current and thus low power consumption but also a feasible and self-powered mode operation.

The invention of the pyro-phototronic effect, which is based on the coupling between three physical fields, pyroelectricity, semiconductor, and photoexcitation, opens a new door regarding the research and development of self-powered high-performance photodetectors. Moreover, it has also been further demonstrated to be a novel light energy harvesting technology. Typically, a voltage bias is required for photodetectors and light energy harvesters. For example, the commonly demonstrated metal-semiconductor-metal photoconductive type photodetector should work with a proper voltage bias. Only with the voltage bias can an electric field be built in the semiconductor to effectively separate and collect photo-generated electrons and holes. Furthermore, considering a single p-n junction-based photodetector (or light energy harvester), generally, it is operated with a negative voltage bias. Because at this bias condition, the depletion region would expand, and the electric field would increase, which is beneficial for the separation and collection of photo-generated electrons and holes. However, first, applying a voltage bias requires external power sources, which might make the whole detection system complex. Second, the dark leakage current could possibly increase when voltage bias is applied. This would cause an increase in energy consumption. Considering these two factors together, developing an energy-saving self-powered system without the need for external power sources is highly desirable, especially for the pyro-phototronic effect. More importantly, the pyro-phototronic-effect-induced performance enhancement is usually the most distinctive when the photodetector operates in a self-powered mode.

Since its invention in 2015 [19], the pyro-phototronic effect has developed as an effective method for improving photodetector’s performances and harvesting light energy for nearly ten years. There are already a few other pieces of literature reviewing the pyro-phototronic effect [20,21,22,23,24,25,26]. Most of these literature reviews focus on the ZnO material-based photodetectors [20,21,22,23], from the conventional device structures to the pyro-phototronic-effect-enhanced self-powered ones. In addition to ZnO, other materials could also present good pyro-phototronic effects and hence, are necessary to be summarized and reviewed. Meanwhile, for the other three literature reviews about the pyro-phototronic effect [24,25,26], they not only discuss the coupling between the pyro-phototronic effect and other effects like piezo-phototronic and localized surface plasmon resonance but also focus on different materials, including ZnO, TiO_2_, CdS, SnS, and so on. In addition to the aspects of materials, the nanostructure and dimension of materials are also very important, as the origin of the pyro-phototronic effect is light-induced transient temperature variation. The absorption and heating ability of incident light could be distinctively different when the same material with different nanostructures and dimensions is utilized. Therefore, it is also necessary to summarize and review the pyro-phototronic effect from the perspective of materials with different nanostructures and dimensions, from 0D quantum dots and 1D nanowires/nanorods to 2D nanoflakes/nanocrystalline thin-films. Moreover, the pyro-phototronic effect has also been demonstrated as an effective method to efficiently harvest light energy and improve the performance of solar cells, and thus needs to be reviewed.

In this review, first, the detailed basic concept and working mechanism of the pyro-phototronic effect are introduced. Then, the recent progress of the pyro-phototronic effect in advanced photodetectors and novel light energy harvesting are thoroughly summarized from the materials’ different dimensions and diversities points of view. Furthermore, the coupling between the pyro-phototronic effect and the piezo-phototronic effect is also reviewed. Finally, the research progresses of the pyro-phototronic effect is summarized, and future perspectives are proposed.

## 2. Fundamentals of Pyro-Phototronic Effect

**Figure 1:**
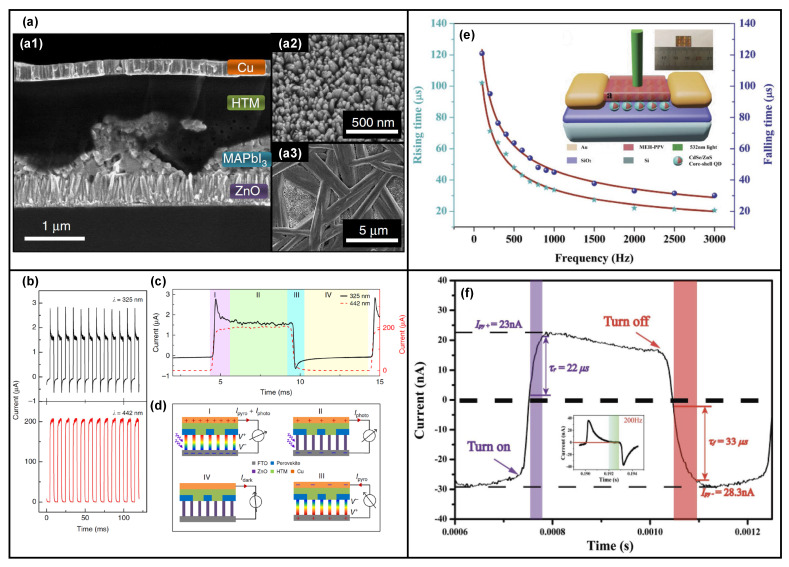
(**a**) SEM images of the as-fabricated perovskite/ZnO nanowires heterojunction; (**a1**) Cross-sectional view of the heterojunction, clearly showing the separate layers; (**a2**) Top view of the as-grown ZnO nanowires; (**a3**) Top view of the spin-coated perovskite on ZnO nanowires; (**b**) The transient photocurrent of the heterojunction in response to 325 and 442 nm laser illuminations, clearly presenting photocurrent peaks while the illumination turns on and off only in 325 nm condition; (**c**) The enlarged transient photocurrent from (**b**) to show detailed differences in transient photocurrent dynamics between 325 and 442 nm laser illuminations; (**d**) The proposed fundamental working mechanism of pyro-phototronic effect; (**e**) The rising and falling times as a function of chopper modulating frequency of the 0D CdSe/ZnS core-shell QD based device. It clearly shows a decreasing trend with increasing frequency. The inset is a schematic of the 0D CdSe/ZnS core-shell QD-based device; (**f**) The transient photocurrent response of the 0D CdSe/ZnS core-shell QD-based device to 532 nm illumination, showing distinctive photocurrent peaks while the light turns on and off. Reproduced with permission from [27]. Copyright 2018 Wiley.

The pyro-phototronic effect was first explored in a perovskite/ZnO nanowires heterojunction [19], where perovskite was spin-coated on the hydrothermally grown ZnO nanowires, followed by spin-coating hole transport material and depositing Cu metal contact electrode, as clearly shown in Figure 1a. Afterward, the response characteristics of perovskite/ZnO nanowires heterojunction to UV (325 nm) and visible (442 nm) laser illuminations were carefully examined. The results indicate that the photocurrent peaks only occur when a 325 nm laser is utilized (Figure 1b,c). The influences of perovskite/ZnO nanowires heterojunction’s temperature and voltage bias are further studied to confirm the underlying working mechanism of occurrence of photocurrent peaks, and it is concluded to be the pyro-phototronic effect.

Figure 1d illustrates the detailed working mechanism of the pyro-phototronic effect, in which a four steps photocurrent behavior should generally be observed. Once the incident light illuminates at the perovskite/ZnO nanowires heterojunction, the incident photons are absorbed by the ZnO nanowires, and corresponding electrons and holes are generated, which is from the traditional photovoltaic effect of semiconductor heterojunction. The absorption of the incident photons would also cause a transient temperature variation of the ZnO nanowires, resulting in a large dT/dt variation rate in them. Since ZnO possesses a pyroelectric effect, light-induced transient temperature variation rate dT/dt would, of course, generate a large number of pyroelectric polarization charges at the two surfaces of ZnO nanowires. Correspondingly, as the +*c*-axis orientation of ZnO nanowires points from the bottom FTO electrode to the top perovskite, positive pyroelectric polarization charges are produced at the perovskite/ZnO nanowires interface, and negative ones are produced at the ZnO nanowires/FTO interface. Noticeably, the direction of pyroelectric potential aligns with the photovoltaic-effect-induced photocurrent I_photo_ flowing in the outside circuit; thus, a transient photocurrent peak caused by the pyroelectric effect I_pyro_ is further introduced. As a result, the total photocurrent that could be observed in the outside circuit is the effective combination of I_photo_ and I_pyro_, which is labeled as I_pyro+photo_ = I_pyro_ + I_photo_ in later research, and this is the first step.

With the time UV laser illuminating on the perovskite/ZnO nanowires heterojunction prolonging, the temperature of ZnO nanowires increases to saturation, i.e., the transient temperature variation rate dT/dt quickly decreases to zero, and hence, the generated pyroelectric polarization charges vanish because the amount of pyroelectric polarization charges is proportional to the parameters dT/dt and pyroelectric coefficient, which is material dependent. Thereby, the pyroelectric-effect-induced photocurrent peak I_pyro_ also quickly decreases to zero, and the total photocurrent returns back to normal I_photo_, which is the second step. Next, when the UV laser illumination on perovskite/ZnO nanowires heterojunction is off, surely the temperature of ZnO nanowires would decrease to some degree, resulting in another transient temperature variation as well as the rate dT/dt, but with opposite polarity. This would again produce a pyroelectric-effect-induced transient photocurrent peak I_pyro_ in the opposite direction, which is the third step. Similarly, the transient temperature variation rate dT/dt would again rapidly recover to zero as the temperature of ZnO nanowires decreases to the initial condition. The total current of perovskite/ZnO nanowires heterojunction then goes back to the general dark leakage current I_dark_, presented as the fourth step. From the working mechanism, it is concluded that the dominant contribution of the pyro-phototronic effect is the light-induced transient temperature variation rate dT/dt. Compared to the response characteristics of perovskite/ZnO nanowires heterojunction to 442 nm laser illumination, these pyro-phototronic-effect-induced photocurrent peaks I_pyro_ not only significantly improve the response speed but also enlarge the photocurrent (from I_photo_ to I_pyro + photo_), as well as the responsivity and detectivity.

## 3. Pyro-Phototronic Effect in Different Dimensional Materials for Photodetectors

### 3.1. 0D Quantum Dots Visible Photodetector

As shown in the inset of Figure 1e, colloidal CdSe/ZnS quantum dots with zero-dimension (0D) and wurtzite structure were synthesized and then spin-coated on the patterned Au electrodes on the SiO_2_/n^+^-Si substrate to form the Au/colloidal wurtzite CdSe/ZnS quantum dots/Au (Au/CWQDs/Au) edge-contact-structured photodetector [27]. This Au/CWQDs/Au photodetector presents a good response to 532 nm laser illumination and obvious photocurrent peaks while the laser turns on/off, as shown in Figure 1f. Thanks to the contribution of the pyro-phototronic effect of colloidal wurtzite CdSe/ZnS QDs, the photocurrent, photoconductive gain, and responsivity are all improved. Further, the response speed is enhanced when the modulating frequency increases to 3000 Hz, with a rise time of about 20 μs and a fall time of about 30 μs (Figure 1e). This work demonstrates that the pyro-phototronic effect could also be realized in 0D QDs as the pyroelectric materials are properly processed to form 0D QDs structure, greatly expanding the material choices regarding the realization of the pyro-phototronic effect.

### 3.2. 1D Nanowires/Nanorods Photodetector

Unlike the 0D QDs-based photodetector, nanomaterials with one-dimensional (1D) structures, especially the nanowires and nanorods, are very well welcomed and commonly seen in the research of the pyro-phototronic effect. For instance, 1D ZnO nanowires own a lot of advantages, including but not limited to facile synthesis methods, low cost, environmentally friendly, excellent optoelectronic properties, and so on. Most importantly, 1D ZnO nanowires are proven to have also good piezoelectric and pyroelectric properties, making them a promising candidate for piezo-phototronic and also pyro-phototronic-related research. Schottky junction/heterojunctions have been reported based on the combination of 1D ZnO nanowires and different metal contact/semiconductors.

**Figure 2:**
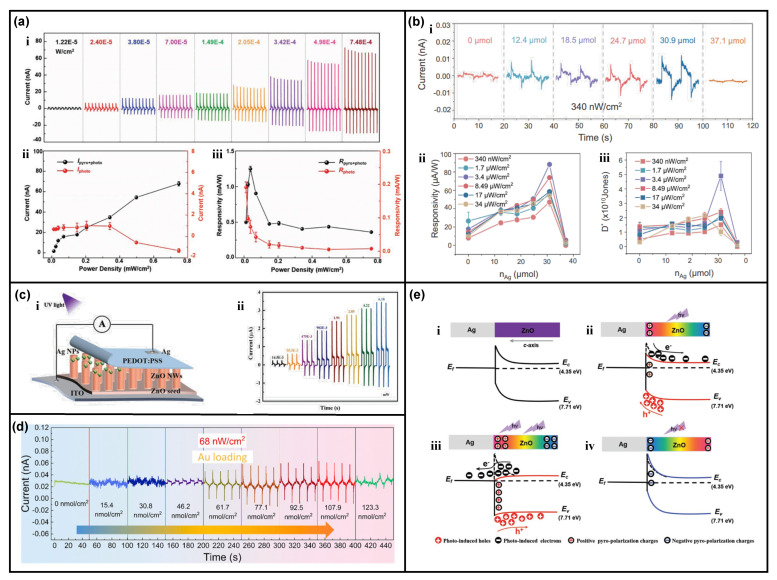
Photoresponse of photodetectors based on metal–ZnO contact. (**a**) Pyro-phototronic-effect-enhanced performances of the self-powered ZnO/Ag Schottky junction photodetector. (**i**) I–t characteristics of pyro-phototronic effect-based photodetector under 325 nm illuminations with different power densities; (**ii**) The transient short-circuits current and the steady-state current as a function of the power density; (**iii**) The corresponding photoresponsivity R_pyro+photo_ and R_photo_ as a function of the power density. Reproduced with permission from [28] Copyright 2018 Wiley; (**b**) The LSPR-enhanced performance of Ag–ZnO photodetectors on UV detection. (**i**) The I–t characteristic curves of Ag–ZnO photodetectors with different loadings of Ag NPs; (**ii**) The responsivity of the Ag–ZnO photodetectors changed as the loading of Ag NPs increases under different power densities; (**iii**) The detectivity of the Ag–ZnO photodetectors varied as the loading of Ag NPs increased under various power densities. Reproduced with permission from [29] Copyright 2022 Wiley; (**c**) Structure and I–t characteristics of the self-powered Ag NPs/ZnO nanowires/PEDOT:PSS photodetector under 325 nm light with various power intensities. Reproduced with permission from [30] Copyright 2022 Wiley; (**d**) The I–t dynamic response characteristics of Schottky junction based on Au NPs@ZnO NW under the illumination of 325 nm light with a power density of 68 nW/cm^2^. Reproduced with permission from [31] Copyright 2022 Wiley; (**e**) Energy band diagrams of the flexible self-powered ZnO/Ag Schottky junction under the conditions of (**i**) dark; (**ii**) the weak illumination; (**iii**) the strong illumination; and (**iv**) withdrawing illumination. Reproduced with permission from [28] Copyright 2022 Wiley.

The Schottky junction was made from 1D ZnO nanowires and Ag metal electrodes on a flexible ITO/PET substrate [28]. In this kind of flexible Ag/ZnO nanowires Schottky junction, the holding conditions could be different; the device could suffer from constant strain or constant stress. Under different mechanical conditions, the contribution of the pyroelectric effect from the ZnO nanowires could be different. When the Ag/ZnO nanowires Schottky junction is fabricated on a hard substrate, e.g., ITO/glass, the constant strain condition should be considered, and then the primary pyroelectric effect is dominant, which is just as similar as the typical pyro-phototronic effect as demonstrated in other works. When the Ag/ZnO nanowires Schottky junction is fabricated on a flexible/soft substrate, e.g., ITO/PET, the constant stress condition should be considered, and then the secondary pyroelectric effect could not be neglected. As shown in Figure 2a, under the 325 nm laser illumination, the pyroelectric polarization charges are induced by the primary pyroelectric effect inside the ZnO nanowires, presenting a transient photocurrent I_pyro + photo_. Simultaneously, the temperature variation of ZnO nanowires would lead to thermal deformation of ZnO nanowires themselves, producing a relatively persistent pyroelectric potential originating from the relative persistent pyroelectric polarization charges of the same polarity at the Ag/ZnO nanowires Schottky junction and hereby lowering the local Schottky barrier height [28]. Once the illumination power density increases strong enough, the Schottky contact between Ag and ZnO nanowires could even be transformed into Ohmic contact, resulting in the reverse flowing of electrons from ZnO nanowires into Ag, i.e., a photocurrent with reverse direction is observed, as illustrated in Figure 2e. This kind of transient photoresponse of Ag/ZnO nanowires Schottky junction is caused by the cooperation of primary and secondary pyroelectric effects, named the comprehensive pyro-phototronic effect, providing a much clearer understanding of the pyro-phototronic effect [28].

Not only the Ag metal could be utilized to form an Ag/ZnO nanowires Schottky junction, but Ag or Au nanoparticles could also be applied to ZnO nanowires for decoration, forming the Schottky junction and introducing the localized surface plasmon resonance (LSPR) at the same time [29,31]. The ZnO nanowires are grown through the hydrothermal method on FTO/glass substrate, and then Ag or Au nanoparticles are decorated onto the ZnO nanowires by sputtering and careful control of sputtering parameters. Compared to the ZnO nanowires-based photodetector without Ag nanoparticles, the Ag nanoparticle decorated Ag/ZnO nanowire Schottky junction photodetector’s performance is greatly improved, as shown in Figure 2b, and it could even respond to 340 nW/cm^2^ 325 nm UV laser illumination. By the contributions from LSPR and pyro-phototronic effect together, the responsivity and detectivity reach 0.0882 mA/W and 4.9 × 10^10^ Jones, respectively [29]. For the Au nanoparticle decorated Ag/ZnO nanowire Schottky junction photodetector, it could even respond to 68 nW/cm^2^ 325 nm UV laser illumination, with responsivity and detectivity of 0.485 mA/W and 2.749 × 10^11^ Jones, respectively (Figure 2d) [31]. These results present the huge potential of the combination of LSPR and the pyro-phototronic effect, especially for the demonstrated Ag/ZnO nanowires Schottky junction. Another PEDOT:PSS/ZnO nanowires heterojunction photodetector’s performances are also greatly improved after the decoration of Ag nanoparticles due to the introduced plasmonic effect (Figure 2c), further proving the effective combination of LSPR and pyro-phototronic effect [30].

**Figure 3:**
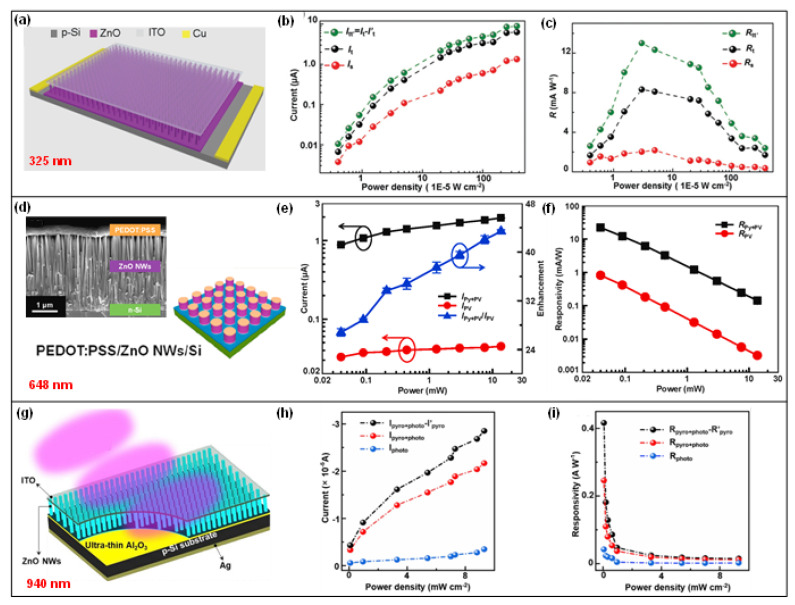
Pyro-phototronic-effect-enhanced performances of UV sensors based on p-Si/n-ZnO under different wavelengths. (**a**) Structure of p-Si/n-ZnO UV sensors; (**b**,**c**) The absolute transient (I_t_, in black), absolute stable (I_s_, in red), and relative transient (I_tt′_, in olive) currents, and the corresponding photoresponsivity change with the power densities. Reproduced with permission from [32]. Copyright 2016 Wiley; (**d**) Structure and scanning electron microscopy images of the p-PEDOT:PSS/n-ZnO nanowires/n-Si tri-layer heterojunction; (**e**) The specific photocurrents I_Py+PV_ and I_PV_ and the enhancement factor at each illuminant power; (**f**) The specific responsivities R_Py+PV_ and R_PV_ at each illuminant power. Reproduced with permission from [37]. Copyright 2020 Elsevier; (**g**) Structure characterizations of the p-Si/Al_2_O_3_/n-ZnO nanowires heterostructured near-infrared photodetector; (**h**) The variation of steady photos, absolute transient and relative transient currents with the power density; (**i**) The corresponding photoresponsivity as a function of the power density. Reproduced with permission from [38]. Copyright 2020 Elsevier.

In addition to the Ag/ZnO nanowires Schottky junction, a pyro-phototronic-effect-enhanced Si/ZnO nanowires heterojunction-based photodetector is also well studied [32,33,34,35,36,37,38,39]. As shown in Figure 3a, ZnO nanowires could be easily grown on the Si substrate to form the Si/ZnO nanowires heterojunction [32]. The as-fabricated Si/ZnO nanowires heterojunction photodetector presents an excellent response to 325 nm laser illumination at reverse bias. It also functions well in the self-powered mode, and obvious pyro-phototronic-effect-induced photocurrent peaks occur in the transient response I–t curves, demonstrating the successful improvement of Si/ZnO nanowires heterojunction photodetector’s performances by the pyro-phototronic effect (Figure 3b). The responsivity is enhanced by nearly 600% (Figure 3c), and the response times, including both rise and fall edges, are reduced to around 20 μs. Unlike the proposed working mechanism of the pyro-phototronic effect in the first literature in 2015, in this Si/ZnO nanowires heterojunction photodetector, the underlying working mechanism mainly focused on the expansion of the depletion region near the Si/ZnO interface. As the ZnO nanowires are grown on the Si substrate, its +*c*-axis orientation points from the bottom Si substrate to the top ITO contact electrode. Once the light is illuminated on the Si/ZnO nanowires heterojunction photodetector, negative pyroelectric polarization charges would be generated at the Si/ZnO interface, repelling the electrons inside ZnO away from the Si/ZnO interface and thus expanding the depletion region of ZnO. Consequently, more incident UV photons could be absorbed, and more electrons and holes are excited and separated, contributing to the photocurrent. Though the working mechanism of the pyro-phototronic effect in Si/ZnO nanowires heterojunction photodetector is a little different from the first presented perovskite/ZnO nanowires heterojunction photodetector, pyro-phototronic-effect-induced performance improvement is obviously presented and verified to be repeatable and stable.

The tuning role of chopper frequency on the pyro-phototronic effect in Si/ZnO nanowires heterojunction photodetector is carefully investigated [33]. The results show that the optimal operating frequency is strongly dependent on the illumination power density and applied voltage bias. When the power density increases from dozens of μW/cm^2^ to a few mW/cm^2^, the optimized chopper frequency also increases from 50 to 200 Hz. While the bias is varied, the corresponding optimal frequency would also vary. The temperature dependence of the pyro-phototronic effect in Si/ZnO nanowires heterojunction photodetector is also reported [34]. When the temperature is cooled down to 77 K, the photocurrent response is significantly improved by about 1300%. If the temperature is kept at room temperature (300 K), the response improvement due to the pyro-phototronic effect is only about 500%. Additionally, the influences of surface/interface states and length of ZnO nanowires with a very large surface-to-volume ratio on the pyro-phototronic effect in Si/ZnO nanowires heterojunction photodetector are systematically studied. It is found that by introducing UV irradiation, the surface/interface states of ZnO nanowires could be greatly modified and hence strengthen the pyro-phototronic effect [35]. For the length of ZnO nanowires and thickness of the Si substrate, the results generally present a trend of a better pyro-phototronic effect observed with thinner Si and short ZnO nanowires [36,37]. To be specific, the ZnO nanowires length’s effect should be considered to be more complex and need optimization case by case as it is strongly related to the ZnO nanowires growth method and parameter control [39]. Moreover, an insulating layer of Al_2_O_3_ could still be added to form Si/Al_2_O_3_/ZnO nanowires tunneling heterojunction photodetector [38]. As clearly shown in Figure 3g–i, the pyro-phototronic-effect-induced performance enhancement is also proved to be effective in the tunneling heterojunction photodetector.

Pyroelectric polarization charges are generated at both surfaces of ZnO nanowires, where positive ones locate at the +*c*-axis orientation surface and negative ones at the other side surface if the temperature variation rate dT/dt is positive, which is similar to the piezoelectric polarization charges produced in ZnO nanowires but decided by the applied strain [40,41]. Typically, only one interface of ZnO nanowires contacts with another metal or semiconductor material to form the Schottky junction or heterojunction. This means that only half of the pyroelectric polarization charges are efficiently utilized. To utilize the pyroelectric polarization charges with both polarities more efficiently, a tri-layer PEDOT:PSS/ZnO nanowires/Si heterojunction photodetector is demonstrated, as shown in Figure 3d [37]. By constructing two hetero-interfaces, all the pyroelectric polarization charges inside ZnO nanowires generated by the light-induced pyroelectric effect are efficiently used to modulate the performance. With the help of such an efficient application of the pyro-phototronic effect, the photocurrent is enhanced about 30 to 40 times, and the responsivity is improved from ~0.8 mA/W to ~22 mA/W in response to 0.04 mW 648 nm laser illumination (Figure 3e,f). Furthermore, for this tri-layer PEDOT:PSS/ZnO nanowires/Si heterojunction photodetector, systematic experimental results show that short ZnO nanowires as the intermediate layer is preferred, which provides coincident evidence on the influences of ZnO nanowires’ length.

**Figure 4:**
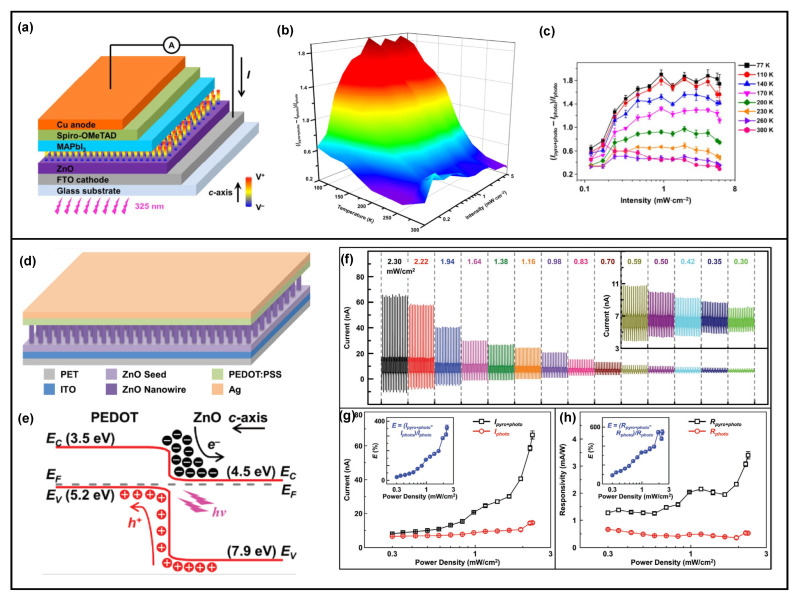
Pyro-phototronic-effect-enhanced photodetectors with heterostructure formed by ZnO and organic materials. (**a**) Schematic demonstration of the structure and operating mechanism of self-powered ZnO/perovskite heterostructured photodetectors; (**b**) 3D surface plot depicting the output-signal enhancement caused by the pyro-phototronic effect under different temperatures and light intensities; (**c**) Output-signal enhancement caused by the pyro-phototronic effect under different UV light intensities, with the temperature ranging from 77 to 300 K. Reproduced with permission from [42]. Copyright 2016 Springer; (**d**) Schematic demonstration of the structure of the PEDOT:PSS/ZnO heterojunction photodetector; (**e**) Energy band diagram of the PEDOT:PSS/ZnO heterojunction; (**f**) Transient characteristics of the pyro-phototronic-effect-enhanced photodetector under 325 nm UV laser with different power densities; (**g**) The pyro-phototronic-effect-induced I_pyro+photo_ and the photovoltaic-effect-induced I_photo_ as a function of the power density; (**h**) The corresponding R_pyro+photo_ and R_photo_ as a function of the power density. Reproduced with permission from [43]. Copyright 2017 Wiley.

In addition to the commercial Si material, other semiconductor materials, such as perovskite, organic semiconductors, NiO, SnS, ZnTe, and CuO, have also been reported to form heterojunctions with ZnO nanowires and their pyro-phototronic effects are investigated [30,37,39,42,43,44,45,46,47,48,49,50,51,52]. The temperature dependence of the pyro-phototronic effect was carefully investigated in the heterojunction shown in Figure 4a, formed by MAPbI_3_ spin-coated on the ZnO nanowires [42]. At low temperatures, the light-induced heating ability is improved, and hence the transient temperature variation rate dT/dt increases, leading to a more obvious photocurrent enhancement by the pyro-phototronic effect, from only ~28% at 300 K to ~170% at 77 K, as illustrated in Figure 4b,c. The charge transfer process and time constant of the dynamic response process are thoroughly studied to reveal the improved pyro-phototronic effect at low temperatures. Similar to perovskite materials, organic semiconductors are also promising candidates to form a heterojunction with ZnO nanowires due to the feasible spin-coating deposition method. A P3HT/ZnO nanowires heterojunction photodetector for self-powered UV detection is demonstrated [44]. With the help of the pyro-phototronic effect, a responsivity of 125 μA/W and detectivity of 3.7 × 10^7^ Jones are achieved at a 0.84 mW/cm^2^ 365 nm illumination. The performance could be further improved by ~20% by cooling down the temperature, which is attributed to the thermo-phototronic effect. These interesting results further provide a possible coupling between the pyro-phototronic effect and the thermo-phototronic effect. In another PEDOT:PSS/ZnO nanorods heterojunction photodetector, the influences of Cl doping in ZnO nanorods on the pyro-phototronic effect is well studied [45]. The experimental results indicate that the Cl-doped ZnO nanorods offer more carriers and hence improve the response to 365 nm illumination by about 330% through the pyro-phototronic effect, as compared to the pristine ZnO nanorods. Our group also reported a PEDOT:PSS/ZnO nanowires heterojunction photodetector and successfully demonstrated the pyro-phototronic-effect-enhanced photocurrent and responsivity to 325 nm laser illumination, as clearly shown in Figure 4d–h [43].

**Figure 5:**
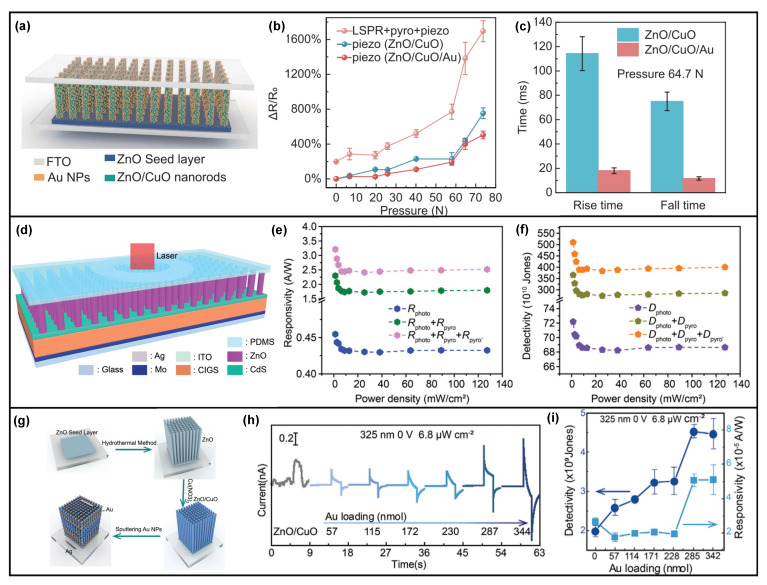
(**a**) Device structure and basic characterization of the self-powered p-CuO/n-ZnO/Au NPs photodetector; (**b**) Δ*R*/*R*_0_ as a function of different applied pressure for quantitatively comparing the piezo-phototronic effect and the comprehensive effect induced responsivity; (**c**) Response time decrease for coupled LSPR-inspired pyro-phototronic effect and piezo-phototronic effect. Reproduced with permission from [50]. Copyright 2022 Wiley; (**d**) Structural diagram of the Cu(In,Ga)Se_2_ multilayer heterojunction photodetector; (**e**,**f**) Responsivities and detectivities as a function of the power density. Reproduced with permission from [51]. Copyright 2022 Wiley; (**g**) Schematic of the preparation process for vertically aligned ZnO/CuO/Au NPs core/shell nanorods photodetector device; (**h**) I–t characteristics of CuO/ZnO photodetectors and CuO/ZnO/Au NP photodetectors with variously loading Au from 57 to 344 nmol under 325 nm illumination; (**i**) Responsivity and detectivity of CuO/ZnO photodetectors and CuO/ZnO/Au NP photodetectors with various Au loading from 57 to 344 nmol under 325 nm illumination. Reproduced with permission from [49]. Copyright 2021 Wiley.

Besides, many researchers observe the photocurrent polarity control by incident light wavelength in self-powered ZnO nanowires-based heterojunction photodetector, and the performances could also be improved by the pyro-phototronic effect [46,47]. By inserting a NiO layer in between ZnO nanowires and Si and applying different voltage biases to the as-fabricated device, the photocurrent polarity presents totally different results, showing excellent light wavelength selectivity [46]. Another ZnO nanowires/SnS heterojunction photodetector also presents similar results; the photocurrents in response to 365/450 nm incident light and that to incident light with a wavelength ranging from 550 to 880 nm own opposite polarity, i.e., the photocurrent polarity could be well controlled by light wavelength [47]. The underlying mechanism is carefully studied and contributed to the coupling between the pyro-phototronic effect in ZnO nanowires and the thermo-phototronic effect in SnS. In addition, CuO and CuBO_2_ have also been reported to form heterojunction photodetectors with ZnO nanowires [49,50,52], and their pyro-phototronic-effect-induced photocurrent enhancement is still observed, as shown in Figure 5a–c,g–i, further demonstrating the suitability of ZnO nanowire-based heterojunction photodetector in pyro-phototronic effect. Some interesting results have been found that by applying pressure to the ZnO nanowires/CuO heterojunction, the responsivity and response time could be further improved (Figure 5b,c) [50], which should be attributed to the coupling between the pyro-phototronic effect and the piezo-phototronic effect, and will be discussed later in Section 5. Further, a Cu(In,Ga)Se_2_ (CIGS)/CdS/ZnO nanowires multilayer heterojunction was reported, and its responses to 405–1064 nm illuminations were systematically investigated [51], as shown in Figure 5d–f, presenting great performance enhancement by the pyro-phototronic effect. For the response to spectral band ranging from 405 to 1064 nm, with the help of the pyro-phototronic effect, a maximum responsivity of ~5 A/W, detectivity of ~8 × 10^12^ Jones, and minimum response time of ~70/80 μs, have been achieved.

In addition to 1D ZnO nanowires, 1D CdS nanowires are also promising candidates for the pyro-phototronic effect [53,54,55,56]. Prof. Yejing Dai reported a flexible Si/CdS nanowires heterojunction photodetector and successfully demonstrated its ultra-broadband response to light wavelength ranging from 325 to 1550 nm based on the pyro-phototronic effect in CdS nanowires [53]. The Si substrate was first chemically etched to a flexible Si thin film, and then pyramid structures were further realized via chemical etching. Then the CdS nanowires were hydrothermally grown on the pyramid-structured Si to form the flexible Si/CdS nanowires heterojunction photodetector. This special device surely exhibited perfect pyro-phototronic-effect-modulated responses to 325 nm and 1060 nm lasers, which were reasonable since Si itself could absorb the corresponding photons and generate photo-excited carriers. More interestingly, this special device amazingly presented photocurrent responses to a 1550 nm laser, which was not expected at first. After a careful evaluation, these interesting results are found to be originated from the pyro-phototronic effect in Si/CdS nanowires heterojunction. When the 1550 nm laser illuminates on the device, though there are absolutely no photo-excited carriers generated due to low photon energy compared to bandgaps of Si and CdS, pyroelectric polarization charges are induced by the pyroelectric property of CdS nanowires, as they are heated by the incident 1550 nm laser illumination. Consequently, the light-induced pyroelectric polarization charges effectively modulate the energy band diagram of Si/CdS nanowires heterojunction photodetector and produce peak-like photocurrents in response to a 1550 nm laser. This work is very important in showing the huge potential application of the pyro-phototronic effect, i.e., an approach for photo-sensing below bandgap energy.

**Figure 6:**
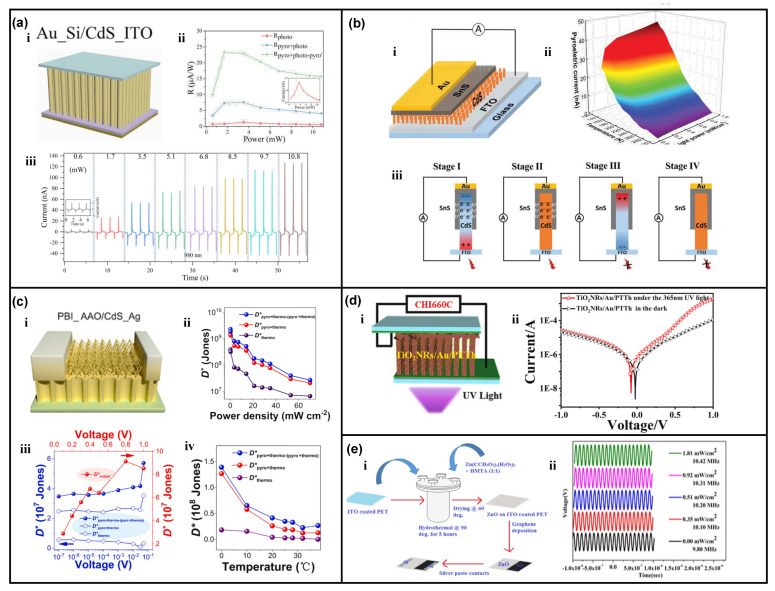
(**a**) Pyro-phototronic-effect-enhanced broadband photodetection based on CdS nanorod arrays. (**i**) Schematic demonstration of the p-Si/n-CdS heterostructure photodetectors; (**ii**) R_pyro + photo_, R_photo_, and R_pyro+photo-pyro′_ of the photodetectors as a function of the power. Inset: Corresponding R_photo_ of the self-powered photodetectors as a function of the power; (**iii**) I–t characteristics of the self-powered photodetectors with different power. Inset: Enlarged I–t characteristics with a 0.6 mW power. Reproduced with permission from [54]. Copyright 2022 Royal Society of Chemistry; (**b**) Structural design and pyroelectric property of SnS/CdS heterojunctions contrived for low-temperature visible photodetectors. (**i**) The schematic structural diagram of the SnS/CdS heterojunction; (**ii**) 3D mapping image of temperature, light power intensity, and pyroelectric current; (**iii**) Diagrammatic sketch of the basic working mechanism on the coupled pyroelectric–photoelectric effects. Reproduced with permission from [55]. Copyright 2020 Wiley; (**c**) Photoresponse characteristics of the self-powered PBI_AAO/CdS_asy-Ag photodetector under 660 nm illumination at different conditions. (**i**) Schematic demonstration of PBI_AAO/CdS_asy-Ag photodetectors; (**ii**) Corresponding R_pyro+thermo−(pyro’+thermo)_, R_pyro+thermo_ and R_thermo_ of the self-powered photodetector as a function of the power density; (**iii**) Corresponding R_pyro+thermo−(pyro’+thermo)_, R_pyro+thermo_, and R_thermo_ of the self-powered photodetectors as a function of the bias voltage from 0 to 50 mV, and the R_output_ of the self-powered photodetectors as a function of the bias voltage from 0.1 to 1 V; (**iv**) Corresponding R_pyro+thermo−(pyro’+thermo)_, R_pyro+thermo_, and R_thermo_ of the photodetector as a function of the background environmental temperature. Reproduced with permission from [56]. Copyright 2022 Elsevier; (**d**) Self-powered TiO_2_ NRs UV photodetectors. (**i**) The schematic diagram of the self-powered ultraviolet detector; (**ii**) I-V curves of TiO_2_ NRs/Au/PTTh heterojunctions. Reproduced with permission from [57]. Copyright 2022 Elsevier; (**e**) Pyro-phototronic nanogenerator based on flexible 2D ZnO/graphene heterojunction and its application in self-powered near-infrared photodetector. (**i**) Schematic of the fabrication procedure for pyrotronic graphene/ZnO diode; (**ii**) Frequency modulation with different intensities showing only ‘ON’ state frequencies. Reproduced with permission from [59]. Copyright 2018 Institute of Physics.

Some other CdS nanowires-based heterojunction photodetectors with performance enhanced by the pyro-phototronic effect have also been reported. Based on the results claimed by Prof. Yejing Dai, another research group fabricated a Si/CdS nanowires heterojunction photodetector by a more facile method: magnetron sputtering [54]. The sputtered CdS nanowires on Si showed good rectification I-V behavior, and the photocurrents to lasers of different wavelengths could be improved by the pyro-phototronic effect, as shown in Figure 6a, indicating the success of magnetron sputtering in the preparation of CdS nanowires for realizing pyro-phototronic effect. The pyro-phototronic effect is further successfully demonstrated in SnS/CdS nanowires (Figure 6b) [55], and polar bear-inspired anodic aluminum oxide (PBI_AAO)/CdS nanowires/Ag heterojunctions (Figure 6c) [56], and the influences of temperature and applied voltage bias are systematically studied. All these results obviously present the promising potential of CdS nanowires in pyro-phototronic effect. Additionally, by using Au nanoparticles as a middle layer in 1D TiO_2_ nanorod/polyterthiophene (PTTh) heterojunction, the photodetector’s response to UV light is further improved due to the pyro-phototronic effect between Au nanoparticles and TiO_2_ nanorods (Figure 6d) [57]. The responsivity is improved from ~0.08 to ~1.9 mA/W. Considering the fact that, TiO_2_ itself does not own pyroelectric properties, the observed pyro-phototronic-effect-induced peak photocurrent and performance improvement are very interesting, possibly being caused by another similar phenomenon, *Alternating Current Photovoltaic Effect*, as claimed by Dr. Haiyang Zou in 2020 with a Si/TiO_2_ heterojunction [58]. Therefore, the peak photocurrent phenomenon and its origin in TiO_2_-based heterojunctions still need in-depth investigations.

### 3.3. 2D Nanoflakes and Nanocrystalline Thin-Films Photodetector

Not only are 1D nanomaterials welcome in the research relating to the pyro-phototronic effect, but two-dimensional (2D) nanomaterials (in the form of nanoflakes and most thin-films) have also been reported to show pyro-phototronic effect. As shown in Figure 6e, 2D ZnO nanoflakes were grown on ITO-coated flexible PET substrate, and then graphene was directly deposited on the as-synthesized 2D ZnO nanoflakes, forming the 2D ZnO nanoflakes/graphene heterojunction diode [59]. The diode presents light intensity controllable modulation of barrier height and good pyro-phototronic-effect-enhanced photocurrent in response to near-infrared (NIR) light illumination. More interestingly, utilizing the pyro-phototronic effect produced pyroelectric potential inside 2D ZnO nanoflakes to modulate the depletion region width, this 2D ZnO nanoflakes/graphene heterojunction diode is adopted as a variable capacitor in a frequency modulator, and the oscillation frequency is successfully modulated. As illustrated in Figure 6e, when the diode is under dark conditions, the frequency is maintained at 9.80 MHz. While the diode is illuminated with 1.81 mW/cm^2^ NIR illumination, the frequency increases to 10.42 MHz. This work not only presents the potential of 2D ZnO nanoflakes in pyro-phototronic-effect-related research, but it also paves the way for applications of pyro-phototronic-effect-modulated diodes in integrated circuits.

**Figure 7:**
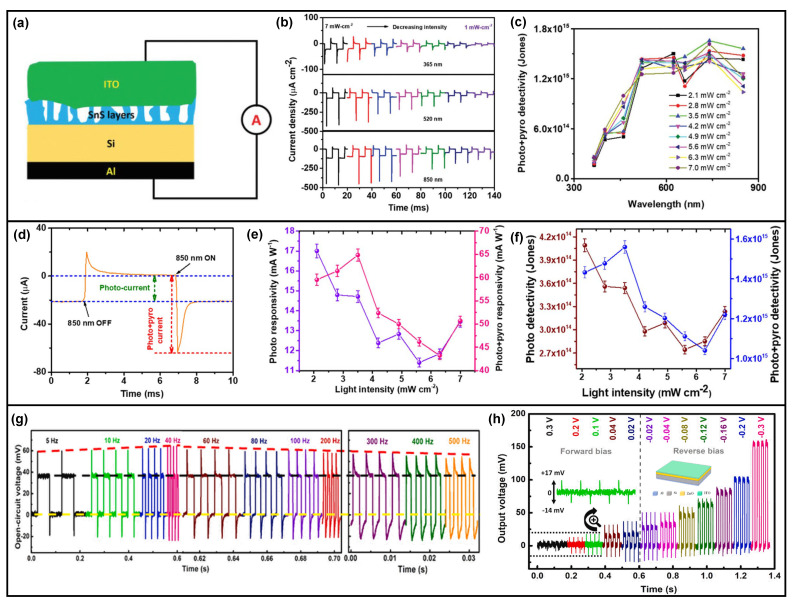
Pyro-phototronic-effect-enhanced photodetectors based on the self-powered ITO/SnS/Si/Al and ITO/ZnO/Si/Al device. (**a**) Cross-sectional schematic; (**b**) Pyroelectric-enhanced photoresponse properties of the SnS/Si device under different LED illumination and self-powered conditions; (**c**) D_ph + py_ as a function of the wavelength for different intensities. Reproduced with permission from [60]. Copyright 2017 Royal Society of Chemistry; (**d**) Transient current density-time characteristics for one cycle with an intensity of 2.1 mW/cm^2^; (**e**,**f**) Responsivity and detectivity for different light intensities. Reproduced with permission from [61]. Copyright 2017 Elsevier; (**g**) The transient response of the pyro-phototronic-effect-based photodetector at different frequencies of 850 nm light and at a fixed intensity of 3.40 mW/cm^2^; (**h**) The influence of bias voltage on the photovoltaic-pyroelectric-coupling-effect-based photodetector under illumination with 3.40 mW/cm^2^ of 850 nm light at 40 Hz. Inset: Schematic demonstration of architecture of p-Si/n-ZnO heterojunction photodetector. Reproduced with permission from [62]. Copyright 2022 Elsevier.

By depositing an SnS thin-film layer via magnetron sputtering on an n-Si substrate, as illustrated in Figure 7a, and optimizing the parameters, Si/SnS heterojunction photodetector has been fabricated [60,61]. As shown in Figure 7a–f, pyro-phototronic-effect-induced photocurrent improvement has been well observed in a broadband wavelength, from UV (365 nm) to NIR (850 nm). At a 7 mW/cm^2^ 760 nm light illumination, thanks to the pyro-phototronic effect, the photocurrent increases from 100 to 470 μA/cm^2^, and the responsivity and detectivity are enhanced to 13 mA/W and 3 × 10^14^ Jones, respectively. Compared to the hydrothermal method to grow 1D ZnO nanowires, magnetron sputtering is a more facile methodology to grow 2D ZnO thin-film, and also, the pyro-phototronic effect has been observed in ZnO thin-film-based heterojunction photodetectors. By magnetron sputtering ZnO thin-film onto commercial Si substrate, Si/ZnO thin-film heterojunction could be fabricated very easily in a cost-effective and time-saving manner [62]. The Si/ZnO thin-film heterojunction shows an excellent response to UV and NIR light illuminations and self-powered operation with the assistance of the pyro-phototronic effect. Both the transient photocurrent and photovoltage exhibit pyro-phototronic-effect-induced peaks. The effects of chopper frequency (Figure 7g) and applied voltage bias (Figure 7h) on the pyro-phototronic effect as a modulation on photovoltage output have been carefully studied, and the results indicate that optimization is required.

**Figure 8:**
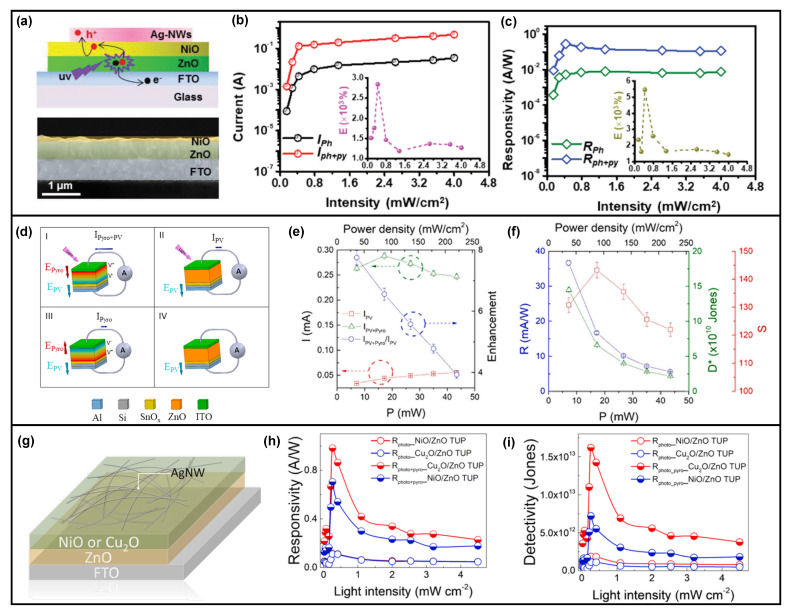
(**a**) Schematic illustration of the working mechanism of the self-powered photodetection based on p-NiO/n-ZnO heterojunction with the corresponding cross-sectional field-emission scanning electron microscopy image; (**b**,**c**) The short-circuit current response and the responsivity response showing the photovoltaic contribution combined with the pyroelectric current at different UV intensities. Reproduced with permission from [65]. Copyright 2019 Wiley; (**d**) Schematic representation of the pyro-phototronic effect in the Al/Si/SnO_x_/ZnO/ITO device for a complete chopper period; (**e**,**f**) Variation of I_Pyro+PV_, I_PV_, and I_Pyro+PV_/I_PV_ and responsivity, detectivity, and sensitivity as a function of laser power density. Reproduced with permission from [69]. Copyright 2021 Elsevier; (**g**) Schematic of transparent ZnO-based photodetector; (**h**,**i**) The UV light (λ = 365 nm, 70 Hz) intensity dependence behavior of responsivity and detectivity for ZnO-based photodetector. Reproduced with permission from [70]. Copyright 2022 Elsevier.

**Figure 9:**
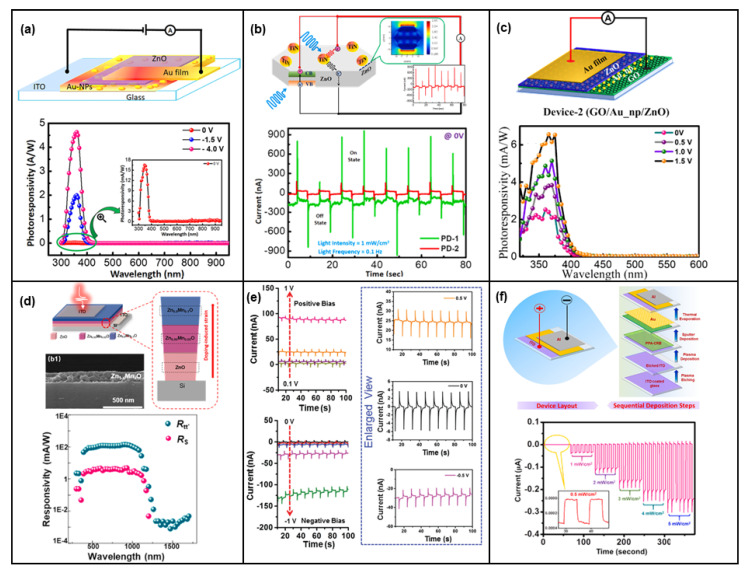
(**a**) The layout and photoresponsivity of the fabricated photodetector having configuration ITO/Au-NPs/ZnO/Au film. Reproduced with permission from [71]. Copyright 2021 Elsevier; (**b**) Structure diagram and comparative I–t response of the device under the illumination of 365 nm light along with the pristine one, which clearly depicts the prominent enhancement in the device performance after the incorporation of TiN nanoparticles in it. Reproduced with permission from [73]. Copyright 2022 Elsevier; (**c**) Architectural structure and photoresponsivity of GO/Au_np/ZnO device at different bias voltages. Reproduced with permission from [72]. Copyright 2022 American Chemical Society; (**d**) Schematic demonstration of the graded p-Si/n-Zn_1−x_Mn_x_O photodetector and corresponding photoresponsivity R_tt’_ and R_s_ change with the excitation wavelength. Reproduced with permission from [74]. Copyright 2022 Elsevier; (**e**) Effect of applied positive and negative bias voltages on the on-off switching characteristics of the photovoltaic-pyro-phototronic coupled plasma-polymerized aniline–crystalline rubrene device under the illumination of switching blue light (450 nm) of frequency 0.1 Hz and intensity 1 mW/cm^2^ along with their enlarged views for applied bias of 0.5 V, 0 V, and −0.5 V. Reproduced with permission from [80]. Copyright 2020 Royal Society of Chemistry; (**f**) Device fabrication process and I–t curve of the plasma polymerized aniline-crystalline Rubrene/Au device for blue light (450 nm) of a frequency of 0.1 Hz showing repetitive on-off cycles with varying intensity at zero applied bias. Reproduced with permission from [81]. Copyright 2021 Elsevier.

In addition to the Si/ZnO structure, heterojunctions formed by ZnO thin film and other metal oxide materials can also exhibit the pyro-phototronic effect [63,64,65,66,67,68,69,70]. As shown in Figure 8a, NiO/ZnO thin-film heterojunction is fabricated on an FTO glass substrate, and both the photocurrent (Figure 8b) and responsivity (Figure 8c) have been largely improved due to the pyro-phototronic effect, with improvements near 3000% and 6000%, respectively [65]. Moreover, Si/SnO_x_/ZnO thin-film heterojunction is demonstrated in Figure 8d to show pyro-phototronic-effect-enhanced photocurrent, with a nearly eight times improvement (Figure 8e,f) [69]. Further, Cu_2_O (Figure 8g–i) [70], Cu_4_O_3_ [66], and V_2_O_5_ [64] are successfully demonstrated with performances being enhanced by the pyro-phototronic effect, showing the versatility of metal oxide/ZnO thin-film heterojunction as a high-performance pyro-phototronic photodetector. Furthermore, both the Au (Figure 9a,c) [71,72] and TiN (Figure 9b) [73] nanoparticles are applicable to ZnO thin-films to induce the pyro-phototronic effect. These nanoparticles could introduce the plasmonic effect and it would effectively couple with the pyro-phototronic effect to further enhance the photocurrent as well as responsivity of the photodetectors, due to the interband transition generated in Au or TiN nanoparticles. As a result of the combination between plasmonic effect and pyro-phototronic effect, the reported photodetectors working in self-powered mode possess responsivity of about dozens of mA/W and a response time of about dozens of microseconds, which is good for ZnO thin-film-based heterojunctions.

Because the bandgaps of semiconductor materials adopted to fabricate heterojunctions are different, the heterojunction photodetectors exhibit different photocurrents and responsivities to light sources with different wavelengths. It is, of course, good when considered from the wavelength selectivity point of view. However, sometimes photodetectors with high and plateau responsivity in ultra-broadband are needed. Prof. Zhaona Wang introduced Mn-graded doping into ZnO thin-film to form a Si/Zn_1-x_Mn_x_O thin-film heterojunction photodetector for photosensing from UV to NIR [74]. As illustrated in Figure 9d, with the graded doping of Mn into ZnO thin-film, the lattice structure of Zn_1-x_Mn_x_O thin-film varies at different positions, and hence, a strain gradient is introduced into the as-synthesized Zn_1-x_Mn_x_O thin-film. This specifically introduced graded strain would produce additional polarization charges as well as the potential to modulate the photo-excited carriers’ dynamic processes. The Si/Zn_1-x_Mn_x_O thin-film heterojunction photodetector demonstrates a plateau responsivity with a value of ~100 mA/W to broadband from 390 to 1100 nm. These findings further indicate the possible coupling between the flexoelectric effect originating from graded strain (or strain gradient) [75,76,77,78,79] and the pyroelectric effect. In addition to inorganic SnS and ZnO films, some organic films also show potential in the pyro-phototronic effect applications. By utilizing a plasma polymerized aniline-crystallization process, rubrene thin-film [80,81] is directly prepared, and then Au nanoparticles are decorated onto it to realize enhanced light absorption. The photocurrent presents distinctive pyro-phototronic-effect-induced peaks while the illumination turns on and off, as shown in Figure 9e,f. It is very exciting that the pyro-phototronic effect is applicable not only in general inorganic materials but also organic materials.

### 3.4. Other Functional- and Dimensional-Materials-Based Photodetectors

**Figure 10:**
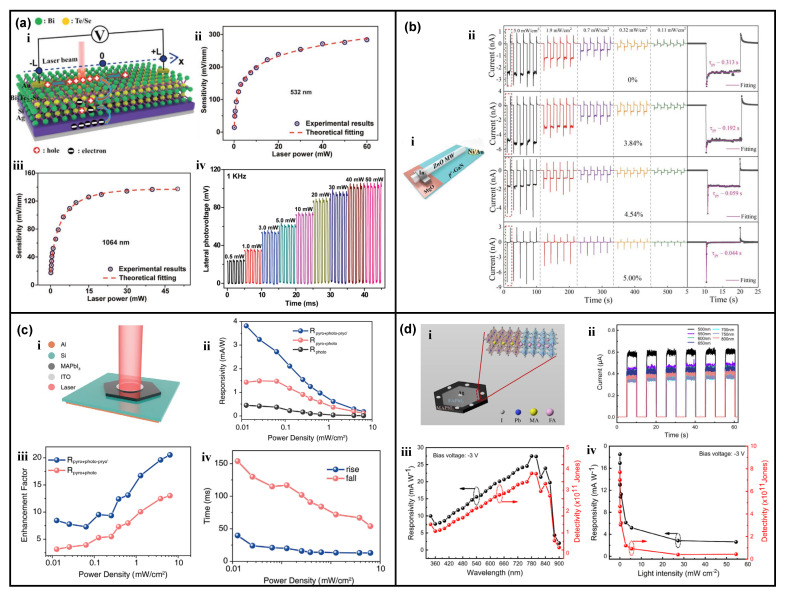
(**a**) Ultraband, large sensitivity position detector based on a Bi_2_Te_2.7_Se_0.3_/Si heterojunction. (**i**) Schematic illustration of the lateral photovoltaic effect in the Bi_2_Te_2.7_Se_0.3_/Si heterojunction; (**ii**,**iii**) The extracted power-dependent sensitivities for 532 and 1064 nm; (**iv**) T-LPV curves of different powers illumination. Reproduced with permission from [82]. Copyright 2019 Wiley; (**b**) Schematic diagrams and I–t curves of MW/p^+^-GaN photodetectors under UV 370 nm illumination with different light intensities, in which the MWs contain different Ga doping concentrations. Reproduced with permission from [83]. Copyright 2022 Wiley; (**c**) MAPbI_3_/Si heterojunction photodetector. (**i**) Schematic diagram showing the structure of MAPbI_3_/Si heterojunction; (**ii**–**iv**) Corresponding R_pyro+photo-pyro’_, R_pyro+photo_, and R_photo_, enhancement factor for (R_pyro+photo-pyro’_)/R_photo_ and R_pyro+photo_/R_photo_ and rise time and fall time as a function of the power density. Reproduced with permission from [85]. Copyright 2021 Wiley; (**d**) The *α*-FAPbI_3_/MAPbI_3_ SC concentric annular lateral heterojunction photodetector. (**i**) Schematic of the *α*-FAPbI_3_/MAPbI_3_ SCs concentric annular lateral heterojunction photodetector; (**ii**) Time-dependent photoresponse under light illumination (0.19 mW/cm^2^) ranging from 500 nm to 800 nm at 3 V bias; (**iii**) Spectral responsivity and detectivity characteristics of the *α*-FAPbI_3_/MAPbI_3_ SC concentric annular lateral heterojunction photodetector with irradiance wavelength ranging from 340 to 900 nm under 3 V bias; (**iv**) Responsivity and detectivity as a function of incident light intensity. Reproduced with permission from [86]. Copyright 2022 Royal Society of Chemistry.

It is also very fascinating to find out that not only the well-known wurtzite structured nanomaterials with different dimensions could present pyro-phototronic effects, but also other functional or single crystalline materials also can. The topological insulator Bi_2_Te_2.7_Se_0.3_ thin-films are prepared on an Si substrate to form the Si/Bi_2_Te_2.7_Se_0.3_ thin-film heterojunction, and the device exhibits self-powered light position sensing ability due to lateral photovoltaic effect (LPE) [82]. Surprisingly, the pyro-phototronic effect is also observed in Si/Bi_2_Te_2.7_Se_0.3_ thin-film heterojunction and could be utilized to modulate both the LPE and time response of the device (Figure 10a). A single Ga-incorporated ZnO (ZnO:Ga) microwire [83,84] together with a p^+^-GaN substrate, forming as GaN/ZnO:Ga microwire heterojunction, also shows good responses to UV 370 nm illumination. By varying the concentration of Ga in ZnO:Ga microwire, the pyro-phototronic-effect-induced modulation could be effectively controlled to be dominant over the photovoltaic effect. As illustrated in Figure 10b, with the increase of Ga’s concentration, the photovoltaic-effect-induced I_photo_ gradually decreases while the pyro-phototronic-effect-induced I_pyro + photo_ increases dramatically. Furthermore, the response time is reduced from hundreds of microseconds to dozens of microseconds. Furthermore, perovskite materials themselves also show the pyro-phototronic effect. Both perovskite MAPbI_3_ single-crystal thin-film/n-Si heterojunction (Figure 10c) [85] and *α*-FAPbI_3_/MAPbI_3_ single-crystal thin-film lateral heterojunction (Figure 10d) [86] could operate in a self-powered mode and exhibit pyro-phototronic-effect-enhanced performances.

**Figure 11:**
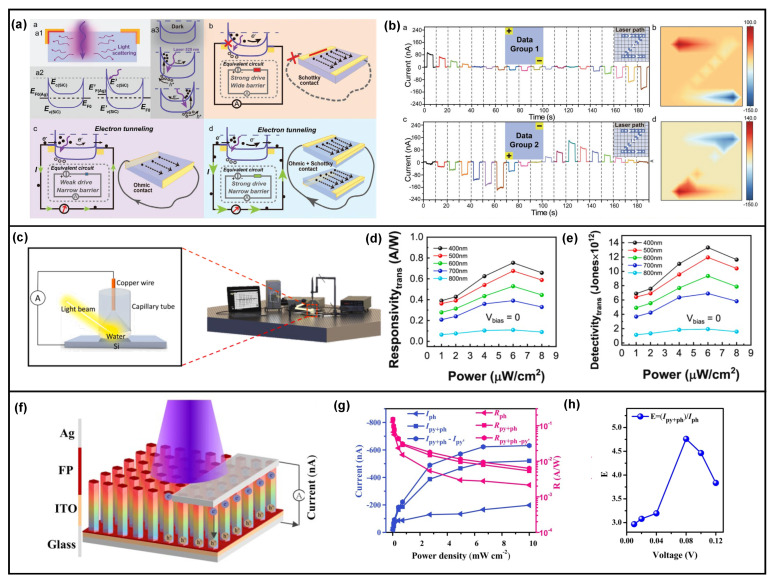
(**a**,**b**) Working mechanism and application of the self-powered 4H-SiC photodetector. Reproduced with permission from [87]. Copyright 2022 Wiley; (**c**) Schematic of the pyroelectric photodetector characterization setup; (**d**,**e**) The responsivity and detectivity of the Si/water device under different light wavelengths as a function of light intensity at zero bias. Reproduced with permission from [88]. Copyright 2022 Elsevier; (**f**) Schematic illustration of self-powered fibrous phosphorus/Ag Schottky junction photodetector; (**g**) I_py+ph_, I_ph_, and I_py+ph−_ I_py’_ and R_py+ph_, R_ph_, and R_py+ph−py’_ of self-powered photodetectors as a function of power density; (**h**) Corresponding enhancement factor E = (I_py+ph_)/I_ph_ of self-powered photodetector as a function of bias voltage. Reproduced with permission from [90]. Copyright 2023 Wiley.

As illustrated in Figure 11a, a self-powered 4H-SiC single crystal UV photodetector has been reported with cooperation between the photo-induced dynamic Schottky effect and the pyro-phototronic effect [87]. The transient-UV-illumination-induced pyro-phototronic effect in 4H-SiC single crystal material dramatically improves the response time to about ten times smaller than the original value. Moreover, it is demonstrated to be able to recognize laser spot position (Figure 11b). Interestingly, even water has been found to have the pyro-phototronic effect. A Si/water heterojunction was fabricated, as shown in Figure 11c, and its performance as a photodetector was measured [88]. The Si/water heterojunction shows responsivity of over 700 mA/W and detectivity over 1 × 10^13^ Jones, respectively, to 400 nm illumination by the pyro-phototronic effect (Figure 11d,e). It is sensitive to the polarization of incident light due to water itself. Additionally, the pyro-phototronic effect could be realized in materials with different structures or morphologies. For example, a porous Si layer [89] and a fibrous phosphorus micropillar array (Figure 11f–h) [90] have been explored to exhibit pyro-phototronic-effect-modulated responses to light illumination. These experimental results and findings extensively expand the choice of materials for pyro-phototronic-effect-enhanced ultra-broadband high-performance photodetectors and provide insights regarding the effect of the material’s structure and surface morphology on light-induced pyro-phototronic effects.

## 4. Pyro-Phototronic Effect in Diversified Materials for Light Energy Harvesting

### 4.1. Light-Triggered Pyroelectric Nanogenerator and Solar Cell

**Figure 12:**
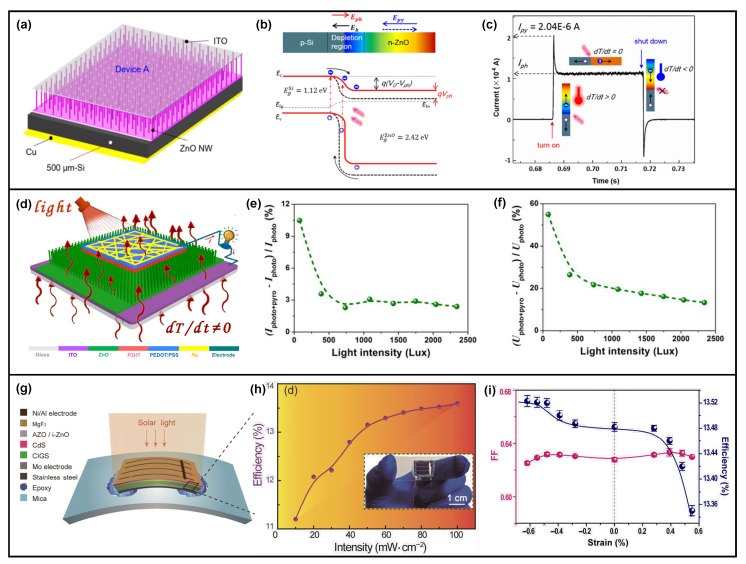
(**a**) Schematic image of the 500 μm p-Si/n-ZnO NW heterostructure devices; (**b**) Photogenerated holes and electrons are separated, leading to the generation of E_ph_; (**c**) One typical cycle of the short-circuit I–t curve. Reproduced with permission from [91]. Copyright 2017 American Chemical Society; (**d**) Illustration of a P3HT/ZnO nanowire array solar cell demonstrating the pyro-phototronic effect; (**e**,**f**) Corresponding enhanced ratios of output currents and voltages by the pyroelectric effect for the P3HT/ZnO solar cell under LED illumination. Reproduced with permission from [92]. Copyright 2016 American Chemical Society; (**g**) Schematic structure of the flexible CIGS solar cell under illumination; (**h**) Solar energy conversion efficiency of the solar cell under AM 1.5G illumination; (**i**) Strain dependence of the solar energy conversion efficiency (*η*) and the fill factor (FF). Reproduced with permission from [93]. Copyright 2018 Springer.

The pyro-phototronic effect has also been established in light energy harvesting. Prof. Xingfu Wang proposed a light-induced heat-triggered pyroelectric nanogenerator based on flexible Si/ZnO nanowires heterojunction for efficient NIR light energy harvesting, as shown in Figure 12a–c [91]. Under dark conditions, the heterojunction is at thermal equilibrium condition, and therefore, the built-in electric field keeps constant. Once the light illumination is introduced, light-induced heating would increase the temperature of ZnO nanowires, and correspondingly, a pyroelectric potential would be produced. This pyroelectric potential either strengthens or weakens the original built-in electric field, depending on the direction, causing a time-dependent variation of the electric field. As a result, this time-varying electric field inside the flexible Si/ZnO nanowires heterojunction then generates Maxwell’s displacement current, converting both the light as photons and thermal energies into electricity. The experimental results indicate that, when reducing the Si thickness down to about 45 μm, the pyro-phototronic-effect-enhanced transient photocurrent peak I_pyro + photo_ could achieve about 1 mA, which is much higher than previously reported value (generally ranging from dozens of nA to a few/dozens of μA). This work paves a potential application of the pyro-phototronic effect in nanogenerators.

Further, the pyro-phototronic effect has been extended to the solar cell to improve the light energy conversion efficiency. A P3HT/ZnO nanowires array heterojunction-based photovoltaic cell (PVC) was demonstrated as shown in Figure 12d–f), and its performance was greatly improved by the pyro-phototronic effect [92]. Moreover, by introducing an external cooling process to further cool down the temperature and potentially induce desired time-dependent temperature variation rate dT/dt, the pyro-phototronic-effect-induced output current and voltage of PVC have been enhanced further, nearly by 20% and 150%, respectively. Another Cu(In, Ga)Se_2_ (CIGS)/CdS/ZnO heterojunction solar cell was proposed to show the pyro-phototronic-effect-induced performance improvement (Figure 12g,h) [93]. The light energy conversion efficiency of the CIGS/CdS/ZnO heterojunction solar cell is improved from 13.48 to 14.23% due to the pyro-phototronic effect by cooling down the temperature from 304 to 275 K at a dT/dt of about 0.6 K/s. Different from the previously discussed pyro-phototronic effect, here the pyro-phototronic effect is introduced by two combined and cooperated methods; one is the typical light-induced heating of materials, and the other is the external cooling or heating to modulate the temperature of the device. These results show that the pyro-phototronic-effect-induced performance modulation of solar cells could be further improved by purposely controlling the temperature variation rate of solar cells through cooling or heating. It is believed that it should be applicable to pyro-phototronic-effect-enhanced high-performance photodetectors. Furthermore, this work still presents the light energy conversion efficiency improvement by further applying compressive strain to the solar cell (Figure 12i), indicating an effective coupling between the pyro-phototronic effect and the piezo-phototronic effect, which will be discussed and summarized later.

### 4.2. Ferroelectric Materials-Based Novel Light Energy Harvesting

**Figure 13:**
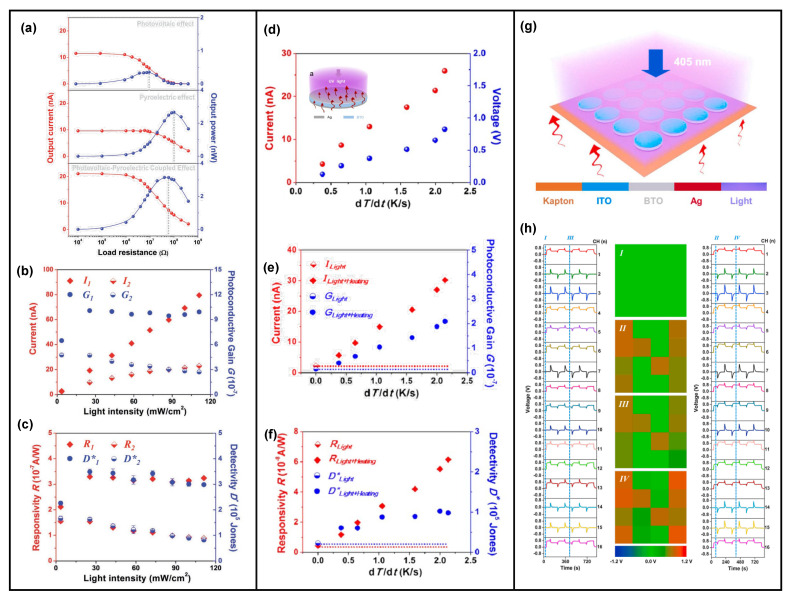
(**a**) Dependence of output current and output power of ITO/BTO/Ag device regarding the photovoltaic effect, the pyroelectric effect, and the photovoltaic–pyroelectric coupled effect on loading resistance under 405 nm illumination; (**b**) Relationship of short-circuit current and photoconductive gain G with light intensity regarding the photovoltaic–pyroelectric-coupled effect and the photovoltaic effect; (**c**) Responsivity and detectivity with light intensity regarding the photovoltaic–pyroelectric coupled effect and the photovoltaic effect. Reproduced with permission from [94]. Copyright 2017 Wiley; (**d**) Dependence of output current and voltage on temperature change rate dT/dt for Ag/BTO/Ag photodetector. Inset: Schematic illustration of Ag/BTO/Ag photodetector in responding to UV light under heating conditions; (**e**,**f**) Relationship of output current and photoconductive gain, responsivity, and detectivity with temperature change rate dT/dt. Reproduced with permission from [95]. Copyright 2017 Elsevier; (**g**) Schematic of the designed flexible photodetector system based on the ITO/BTO/Ag structure; (**h**) V-t curves and mapping images of the self-powered photodetector system under different conditions. Reproduced with permission from [102]. Copyright 2020 Elsevier.

In addition to traditional semiconductor materials, ferroelectric materials also have been proved to be capable of converting light energy into electricity, i.e., ferroelectric materials are potential candidates for light energy harvesting. Thus, owning to their intrinsic ferroelectric properties, the pyro-phototronic effect could be utilized in ferroelectric materials to improve their light energy harvesting ability. Prof. Ya Yang reported ferroelectric BaTiO_3_ (BTO) [94,95,96,97,98,99,100,101,102,103,104] thin-film light energy harvesters could scavenge photon energy. As shown in Figure 13, commercial BTO powder with nanoparticle morphology was utilized to sinter the ferroelectric BTO thin-film, and then ITO and Ag were deposited as top and bottom contact electrodes. By coupling the photovoltaic effect and pyroelectric effect (i.e., pyro-phototronic effect) of BTO thin-film, all the important parameters of a light energy harvester, including photocurrent, photoconductive gain, responsivity, and detectivity, have been largely improved [94]. More importantly, in another BTO-thin-film-based light energy harvester fabricated from BTO nanowires, it is found that, by introducing external heating of the BTO-based device, all the above-mentioned important merits would be further enhanced [95]. With the increase in heating-induced temperature variation rate dT/dt, the enhancement increased, exhibiting a maximum increment of over 1000% in photocurrent, photoconductive gain, and responsivity. Furthermore, the detectivity maximum increment is over 300%. These results not only extend the material choice of the pyro-phototronic effect into ferroelectric materials but also further confirm that the pyro-phototronic effect could be modulated by the device’s temperature controlled by purposely cooling or heating. Based on these distinctive observations and results, a self-powered image mapping system was successfully demonstrated [102]. As shown in Figure 13g,h, a device matrix composed of 16 BTO-based devices (4 × 4) was formed, and it was able to recognize the letter “N” under an external cooling condition, showing great potential for self-powered light position and/or temperature variation mapping.

**Figure 14:**
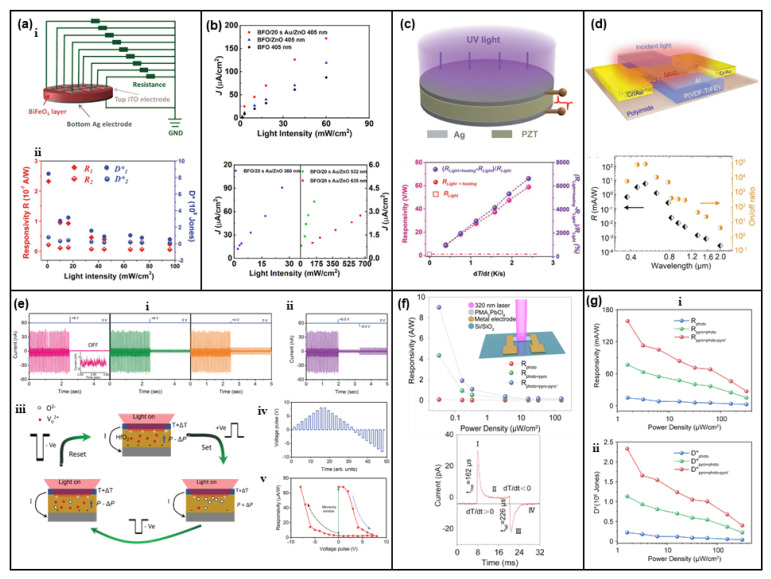
(**a**) ITO/BiFeO_3_/Ag photodetector. (**i**) Schematic diagram of the structure of 3 × 3 ITO/BiFeO_3_/Ag photodetector array; (**ii**) Responsivity and detectivity on the light intensity with regard to photovoltaic–pyroelectric coupled effect and photovoltaic effect based on ITO/BiFeO_3_/Ag photodetector. Reproduced with permission from [105]. Copyright 2017 Wiley; (**b**) Light intensity dependence of photocurrent density of BiFeO_3_ films, BiFeO_3_/ZnO, and BiFeO_3_/20 s Au/ZnO heterostructures under 405, 360, 532, and 635 nm light illumination at zero bias. Reproduced with permission from [106]. Copyright 2021 Elsevier; (**c**) Schematic diagram of Ag/PZT/Ag device and dependence of responsibility on the temperature change rate dT/dt, as well as the corresponding enhancement when Ag/PZT/Ag device, respond to 365 nm light in a heating state. Reproduced with permission from [107]. Copyright 2017 Wiley; (**d**) 3D schematic of the photodetector constructed by a hybrid quasi-freestanding structure of organic ferroelectric poly(vinylidene fluoride–trifluoroethylene) with MoS_2_, photoresponsivity, and on/off photocurrent switching ratio of the photodetector for incident light wavelengths ranging from 375 nm to 2 µm at V_SD_ = 1 V. Reproduced with permission from [108]. Copyright 2019 Wiley; (**e**) Pyro-phototronic memory based on HfO_2_-based self-powered infrared pyroelectric sensor. (**i**) The transient pyro-photoresponse of the device with continuous light pulse illumination before and after applying an electric pulse; (**ii**) The transient pyro-photoresponse of the device with applied positive and negative pulses of the same magnitude; (**iii**) Working mechanism; (**iv**) Shape of the applied voltage; (**ⅴ**) The change of the pyro-photoresponsivity with following the electric pulses as shown in (**iv**). Reproduced with permission from [109]. Copyright 2022 Wiley; (**f**) Ferro-pyro-phototronic effect in monocrystalline 2D ferroelectric perovskite for high-sensitive, self-powered, and stable ultraviolet photodetector. Reproduced with permission from [110]. Copyright 2022, American Chemical Society; (**g**) UV photoresponse performances of the PMA_2_PbCl_4_ photodetector under 320 nm laser illumination at zero bias. (**i**) Corresponding R_photo_, R_pyro+photo_, and R_pyro+photo-pyro′_ as a function of power density; (**ii**) Corresponding D*_photo_, D*_pyro+photo_, and D*_pyro+photo-pyro′_ of the self-powered photodetector as a function of power density. Reproduced with permission from [111]. Copyright 2022 American Chemical Society.

Other than BTO, ferroelectric material BiFeO_3_ (BFO) [105,106] has been verified to show similar pyro-phototronic-effect-enhanced light energy harvesting ability, exhibiting maximum photosensing performance enhancement of over nine times with 0.86 mW/cm^2^ 450 nm light illumination by utilizing light-induced pyro-phototronic effects (Figure 14a). Furthermore, three devices, BFO, BFO/ZnO heterojunction, and BFO/Au/ZnO tri-layer heterojunction, have been compared. The results indicate that BFO/Au/ZnO tri-layer heterojunction produces improved photocurrent compared to BFO/ZnO heterojunction and BFO (Figure 14b). Meanwhile, Au nanoparticles broaden the response wavelength from UV to NIR. The Pb(Zr,Ti)O_3_ (PZT) based pyro-phototronic effect light energy harvester has also been studied, and its performance is also further improved by external heating (Figure 14c) [107].

The intrinsic pyroelectric property of organic ferroelectric material poly(vinylidene fluoride–trifluoroethylene) (P(VDF-TrFE)) has been explored to couple with the photoelectric effect of 2D molybdenum disulfide (MoS_2_), demonstrating an ultra-broadband light energy harvesting from 375 nm to 10 μm, as shown in Figure 14d [108]. This is a novel device design to realize the pyro-phototronic effect. For the spectral range over 2 μm, both P(VDF-TrFE) and MoS_2_ could not respond since their bandgap is much larger. However, the heating ability of IR light is excellent, and thus, the pyroelectric polarization charges, as well as pyroelectric potential, could be induced in P(VDF-TrFE). Then, MoS_2_ is adopted as a channel to read and amplify the pyroelectric-effect-induced photocurrent, finally achieving the response to IR light over 2 μm by pyro-phototronic effect through the novel device design. In addition to the improvement of light energy harvesting, the pyro-phototronic effect in this novel device design further extends the responsive spectral wavelength to an ultra-broad level, from 375 nm to 10 μm, presenting huge potential applications in high-performance light energy harvesting and photodetector.

Ferroelectric hafnium oxide (HfO_2_) material has further been demonstrated as a performance-enhanced light energy harvester and a reconfigurable pyro-phototronic memory in Figure 14e [109]. By purposely applying an electric pulse, the pyro-phototronic-effect-induced photocurrent output could be effectively modulated. The initial photocurrent I_pyro + photo_ is about 40 nA and could be reduced to about only 1 nA by applying a +8 V electric pulse. More importantly, the application of a negative electric pulse could recover the photocurrent I_pyro + photo_, but not capable of recovering back to the initial value. After a careful investigation, the electric pulse reconfigurable pyro-phototronic memory is found to be caused by the migration of oxygen ions inside HfO_2_. Such a migration of oxygen ions upon external electric pulse would modify the effective pyroelectric polarization of HfO_2_, which in turn modulates the pyro-phototronic-effect-induced photocurrent I_pyro + photo_. Furthermore, ferroelectric perovskite PMA_2_PbCl_4_ (Figure 14f,g) [110,111] and some other ferroelectric composites and perovskites [112,113,114,115,116,117] have also been synthesized and explored to show pyro-phototronic-effect-enhanced light energy harvesting performances, presenting the ferroelectric materials’ huge potentials in the application of the pyro-phototronic effect.

## 5. Coupling of Pyro-Phototronic and Piezo-Phototronic Effects

**Figure 15:**
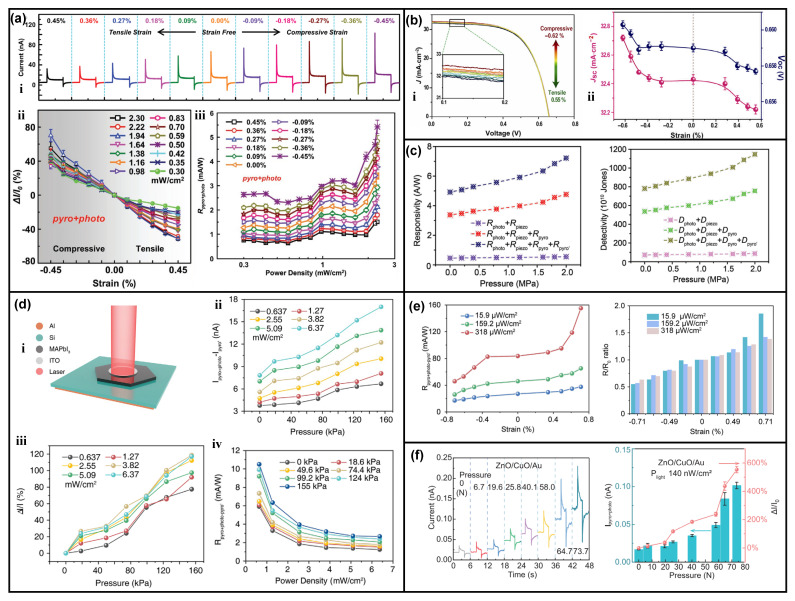
(**a**) The externally applied strain’s effects on the self-powered PEDOT:PSS/ZnO heterojunction. (**i**) Transient I–t characteristics of the pyro-phototronic-effect-enhanced photodetector under 325 nm 2.30 mW/cm^2^ UV illumination with different strains; (**ii**) The relative change of I_pyro+photo_, ΔI/I_0_, as a function of externally applied strain under different power densities; (**iii**) The calculated R_pyro+photo_ as a function of power density under different externally applied strains. Reproduced with permission from [43]. Copyright 2017 Wiley; (**b**) Performance of CIGS solar cell under strains at room temperature. (**i**) J–V characteristics of the solar cell under different strains; (**ii**) Strain dependence of the short-circuit current density (J_SC_) and the open-circuit voltage (V_OC_). Reproduced with permission from [93]. Copyright 2018 Springer; (**c**) Responsivities and detectivities as a function of the pressure for the CIGS heterojunction photodetector. Reproduced with permission from [51]. Copyright 2022 Wiley; (**d**) A self-powered photodetector based on MAPbI_3_ single-crystal film/n-Si heterojunction. (**i**) Schematic diagram; (**ii–iv**) The I_pyro+photo_-I_pyro’_, ∆I/I_0_, and the R_pyro+photo-pyro’_ as a function of pressure toward 780 nm laser illumination under zero bias with different power densities. Reproduced with permission from [85]. Copyright 2021 Wiley; (**e**) R_pyro+photo-pyro′_ and responding strain dependence of enhancement ratio of R_pyro+photo-pyro′_ as a function of applied tensile strain with different incident light power density for the flexible PMA_2_PbCl_4_ photodetector. Reproduced with permission from [111]. Copyright 2022 American Chemical Society; (**f**) Transient I–t characteristics of ZnO/CuO/Au devices under different external pressure and corresponding photocurrent as well as relative change of ΔI/I_0_ under 325 nm illumination. Reproduced with permission from [50]. Copyright 2022 Wiley.

Considering the fact that the pyro-phototronic effect is the coupling between pyroelectricity, semiconductors, and a photoelectric property, where pyroelectric polarization charges are significant, it is possible to further demonstrate multi-fields coupling among the pyro-phototronic effect and the piezo-phototronic effect. In such a manner, polarization charges could be produced by both pyroelectric and piezoelectric effects, i.e., light-induced temperature variation and externally applied strain. In 2017, our group first reported the effective cooperation between the pyro-phototronic effect and the piezo-phototronic effect in an organic/inorganic heterojunction photodetector [43]. The ITO-coated PET flexible substrate was chosen to hydrothermally grow ZnO nanowires, followed by spin-coating poly(3,4-ethylenedioxythiophene)-poly(styrene sulfonate) (PEDOT:PSS), finally realizing the flexible PEDOT:PSS/ZnO nanowires heterojunction photodetector, which is easy to apply strains in both directions (compressive and tensile). The as-fabricated PEDOT:PSS/ZnO nanowires heterojunction photodetector presents good UV response with performance enhanced by the pyro-phototronic effect. Then, by applying different compressive and tensile strains to the device, not only the photovoltaic-effect-induced photocurrent I_photo_ but also the pyro-phototronic-effect-induced photocurrent I_pyro+photo_ have been effectively modulated, as shown in Figure 15a. It is obvious that, with the application of compressive strains, both the photocurrents I_photo_ and I_pyro+photo_ are improved. The underlying mechanism is carefully investigated and contributed to the effective coupling of pyroelectric polarization charges generated by light-induced heating and piezoelectric polarization charges caused by compressive strain.

Afterward, similar results were reported in CIGS/CdS/ZnO heterojunction [51,93] as a solar cell (Figure 15b) and photodetector (Figure 15c). The light energy conversion efficiency, as well as the responsivity and detectivity, could be improved further by the cooperation of the pyro-phototronic effect and the piezo-phototronic effect. Furthermore, the MAPbI_3_/Si heterojunction (Figure 15d) [85], PMA_2_PbCl_4_ single crystal microbelt arrays (Figure 15e) [111], and Au decorated ZnO/CuO nanorods heterojunction (Figure 15f) [50] photodetectors have also been reported as their performances as self-powered photodetectors could be improved further by properly coupling the pyro-phototronic effect and the piezo-phototronic effect. Recently, our group reported a pyramid-structured Si/ZnO nanowires heterojunction photodetector with performance enhancement by the pyro-phototronic effect [118]. The influences of ZnO nanowire’s growth parameters and their surface morphology on the pyro-phototronic effect have been systematically investigated. The results indicate that the surface morphology greatly affects the absorption of incident light illumination and hence, the light-induced temperature variation rate, finally leading to the efficient modulation of the pyro-phototronic effect. Furthermore, when compressive strain is introduced, the photocurrent is reduced due to the coupling of the pyro-phototronic effect and the piezo-phototronic effect. The working mechanism is proposed to be attributed to the possible multi-field coupling among the pyroelectric effect, the piezoelectric effect, and the flexoelectric effect, as the strain distribution in pyramid-structured Si is definitely non-uniform, and thus, a strain gradient occurs, inducing flexoelectric polarization charges together with piezoelectric and pyroelectric polarization charges. The limited literature report fascinating results regarding the multi-field coupling and open a window for possible research toward the cooperation of all kinds of polarization charges, including piezoelectric, pyroelectric, flexoelectric, and ferroelectric approaches.

The performances of devices have been summarized and compared, as shown in Table 1. It is obvious that, for the pyro-phototronic-effect-enhanced photodetectors, the reported 0D CdSe/ZnS quantum dots-based device shows a relatively low responsivity and detectivity. For the 1D nanowires/nanorods-based devices, most of them show short response times (dozens of microseconds), relatively high responsivities (a few to dozens of mA/W), and detectivities (~10^11^ Jones). While for 2D ZnO thin-film-based devices, their performances are similar to 1D ZnO nanowires-based devices. Noticeably, the reported 2D SnS-based devices present very high detectivity (~10^14^ Jones) together with fast response speeds (~dozens of microseconds) and relatively high responsivity (dozens of mA/W). Other functional and dimensional materials show similar performances compared to 1D nanowires/nanorods-based devices. Specifically, more attention should be paid to the reported Si/water heterojunction. Under 400 nm laser illumination and self-powered working mode, it presents a responsivity as high as 754 mA/W and a detectivity as high as ~10^13^ Jones, though the response time is a bit longer (from dozens to hundreds of milliseconds). These results indicate the massive potential of 1D and 2D materials, especially the ZnO and SnS, in pyro-phototronic-effect-enhanced photodetectors. Furthermore, the Si/water heterojunction also opens the door to solution-based heterojunctions for pyro-phototronic-effect-enhanced high-performance photodetectors.

For the pyro-phototronic-effect-enhanced light energy harvesters, the performances of different materials-based devices are comparable to each other. All these demonstrated devices prove that the pyro-phototronic effect does greatly improve light energy harvesting efficiency. Lastly, concerning the coupling between pyro-phototronic effect and other effects, such as the piezo-phototronic effect and localized surface plasmon resonance, it could be observed that the detectivity and responsivity could be enhanced up to ~10^13^ Jones and a few A/W, respectively. These results further validate the potential of the pyro-phototronic effect in high-performance light energy harvesters and the synergistic effect when coupled with other promising effects.

In addition to the pyro-phototronic effect, triboelectric nanogenerators (TENG) and the piezoelectric effect could also be utilized as photodetectors and energy harvesters. Compared to pyro-phototronic-effect-based nanogenerators, triboelectric and piezoelectric nanogenerators are mostly demonstrated for mechanical energy harvesting [9,10,119]. TENG utilizes contact-electrification-induced triboelectric surface charges to induce Maxwell’s displacement current, while piezoelectric nanogenerators (PENG) utilize piezoelectric-effect-induced piezoelectric charges for converting mechanical energy into electrical energy [13]. Interestingly, TENG and PENG could also be used as a photodetector. The working mechanism is mainly based on the resistance variation of nanogenerators or external loads when it is under light illumination [120,121]. Different from TENG and PENG, the pyro-phototronic effect is used as an advanced photodetector or efficient light energy harvester, which is based on the photovoltaic and pyroelectric effects. Therefore, TENG, PENG, and the pyro-phototronic effect could be integrated together to demonstrate a multi-effects-coupled hybrid nanogenerator capable of harvesting and detecting mechanical and light energies and stimulus [122,123].

## 6. Summary and Perspectives

In summary, the recent progress on the pyro-phototronic effect for advanced photodetectors and light energy harvesters has been reviewed and discussed. The fundamental working principle of the pyro-phototronic effect is revealed, which is caused dominantly by the light-induced temperature variation. Various kinds of nanomaterials with different dimensions (from 0D to 2D and even single crystals) have been explored and proven to demonstrate great enhancements by the pyro-phototronic effect in photodetectors and novel light energy harvesters. The coupling of the pyro-phototronic effect and the piezo-phototronic effect has also been reviewed. The pyro-phototronic effect is undoubtedly proved to be an effective methodology for improving the performances of advanced optoelectronic devices in potential applications, e.g., ultrafast photodetection, optical communication, photo-memory, light position recognition and 2D mapping, and so on. Additionally, some issues and perspectives are needed to be pointed out.

First, the fundamental working mechanisms from a device physics perspective of the pyro-phototronic effect require further systematic and in-depth investigation. In the first report on perovskite/ZnO nanowires heterojunction, the photocurrent peak is attributed to the alignment of pyroelectric and photovoltaic potentials. While in most reported Si/ZnO nanowires heterojunctions, it is attributed to the time-dependent variation of depletion region or electric field. Moreover, there is literature reporting pyro-phototronic-effect-like photocurrent peaks in non-pyroelectric materials, and it is found to be the *Alternating Current Photovoltaic Effect*. It is believed that further experiments with careful designs should be carried out on the device physics level to reveal the in-depth working mechanism behind the pyro-phototronic effect in various semiconductors and devices.

Second, though the choice of the material for pyro-phototronic-effect-related research has been explored and expanded to some degree, it is still mainly focused on ZnO, CdS, and traditional ferroelectric materials like BTO, BFO, and PZT. Further, the dimensions are mostly 1D nanowires and 2D thin films. More nanomaterials with different dimensions and diversified functions should be further explored in the pyro-phototronic-effect-related research. For example, the 2D and 0D nanomaterials, topological insulators, functional metal oxides (HfO_2_, and so on), and perovskites.

Third, although the performance of the device is improved several times or even tens of times under the modulation of the pyro-phototronic effect, the figure of merits, such as photocurrent and photoresponsivity, still need to be enhanced for practical applications. The pyro-phototronic-effect-induced photocurrent I_pyro + photo_ generally varies from dozens of nA to a few μA, and the corresponding responsivity ranges from dozens of μA/W to dozens of mA/W. Few devices have achieved photocurrent as large as a few milliamperes and responsivity close to A/W. The response time is also mainly at the scale of dozens of microseconds. Therefore, the device structure and configuration, together with the choice of materials (including synthesis and properties), should be carefully designed and optimized to enhance the photocurrent, responsivity, and response time.

Fourth, it has been shown that the coupling between the pyro-phototronic effect and the piezo-phototronic effect is verified and proven to be an advanced methodology to further improve the device’s performances. In some research, the thermoelectric effect is also proven to be able to couple with the pyro-phototronic effect. Hence, the multi-field coupling among pyroelectric, thermoelectric, piezoelectric, flexoelectric, and ferroelectric effects should have tremendous amounts of attention paid to it. A possible introduction of the magnetoelectric effect and the acoustoelectric effect could be considered. These interesting multi-physics couplings would certainly yield novel functional optoelectronic devices and undiscovered phenomena.

Fifth and finally, ever since the invention of the pyro-phototronic effect, it is mostly studied and demonstrated to improve the performance of photodetectors. There is a lack of literature reporting on the pyro-phototronic-effect-induced improvement in solar cells. More potential applications should be explored, including but not limited to ultra-broadband photodetectors with high-performances, functional photo-memories, solar cells with enhanced light energy conversion efficiency, ultrafast photodetection (lower than the scale of μs), all-optical logic computation and communication, photo-location recognition and 2D mapping, and so on.

## Figures and Tables

**Table 1:** Comparison of performances of pyro-phototronic-effect-enhanced photodetectors and light energy harvesters.

	Structure	Wavelength	Intensity	Bias (V)	Rise Time	Fall Time	Detectivity (Jones)	Responsivity	Ref. No.
Pyro in 0D QDs	CdSe/ZnS core-shell QDs	532 nm	332.5 mW/cm^2^	0	20.6 us	30.1 us	2.43 × 10^6^	2.17 × 10^−6^ A/W	[27]
Pyro in 1D ZnO	ZnO NWs/MAPbI_3_	325 nm	1.9 × 10^−5^ W/cm^2^	0	53 us	63 us	4.00 × 10^10^	26.7 mA/W	[19]
	ZnO NWs/Ag	325 nm	3.8 × 10^−5^ W/cm	0	4.5 ms	3.5 ms	2.70 × 10^9^	1.25 mA/w	[28]
	ZnO NWs/Ag NPs	325 nm	340 nW/cm^2^	0	25.87 ms	/	1.51 × 10^10^	0.047 mA/W	[29]
	ZnO NWs/Au NPs	325 nm	68 nW/cm^2^	0	12.01 ms	12.41 ms	2.75 × 10^11^	0.485 mA/W	[31]
	Ag NPs@ZnO NWs/PEDOT:PSS	325 nm	14.5 × 10^−3^ mW	0	0.1 ms	0.2 ms	6.73 × 10^10^	25.4 mA/W	[30]
	Si/ZnO NWs	325 nm	3.7 × 10^−3^ W/cm^2^	0.04	1.5 ms	1.2 ms	13 × 10^8^	13 mA/W	[32]
		442 nm	1 × 10^−2^ W	0.4	15 us	12 us	/	62 mA/W	
	Si/Al_2_O_3_/ZnO NWs	940 nm	8.4 × 10^−5^ W/cm^2^	0	15 us	21 us	3.68 × 10^12^	0.41 A/W	[38]
Pyro in 1D CuO	ZnO/CuO core–shell NRs with Au NPs	325 nm	68 nW/cm^2^	0	6.3 ms	6.9 ms	3.3 × 10^11^	1.4 × 10^−4^ A/W	[49]
Pyro in 1D CdS	Si/CdS NRs	980 nm	1.7 mW	0	70 us	90 us		23 uA/W	[54]
	CdS NRs/SnS nanoflakes	650 nm	0.08 mW/cm^2^	0	20 ms	18 ms	3.56 × 10^11^	10.4 mA/W	[55]
	PBI_AAO/CdS_asy-Ag	660 nm	0.24 mW/cm^2^	0	0.2 s	4 s	2.3 × 10^9^	4 mA/W	[56]
Pyro in 1D TiO_2_	TiO_2_ NRs/Au/PTTh	365 nm	0.41 mW/cm^2^	0	0.23 s	0.28 s	1.666 × 10^10^	1.894 mA/W	[57]
Pyro in 2D SnS	Si/SnS	760 nm	7 mW/cm^2^	0	12 us	55 us	3 × 10^14^	13 mA/W	[60]
	Si/SnS	850 nm	5 mW/cm^2^	0	12 us	42 us	1.6 × 10^15^	65 mA/W	[61]
Pyro in 2D ZnO	2D ZnO/graphene	780 nm	/	0	90 ms	78 ms	/	2.64 mA/W	[59]
	Si/ZnO heterojunction	850 nm	3.4 mW/cm^2^	0	78 μs	152 μs	3.2 × 10^11^	6 mA/W	[62]
	NiO/ZnO heterojunction	365 nm	0.43 mW cm^2^	0	3.92 μs	8.90 μs	2.75 × 10^11^	0.29 A/W	[65]
	Si/SnO_x_/ZnO	405 nm	36 mW/cm^2^	0	3 μs	2 μs	1.5 × 10^11^	36.7 mA/W	[69]
	ZnO/Cu_2_O	365 nm	0.26 mW/cm^2^	0	42.5 μs	56 μs	1.62 × 10^13^	0.98 A/W	[70]
Pyro in other functional and dimensional materials	Bi_2_Te_2.7_Se_0.3_/Si	532 nm	50 mW	/	58 μs	78 μs	/	/	[82]
	ZnO:Ga MW/GaN	370 nm	0.11 mW/cm^2^	0	/	0.044 s	/	16 mA/W	[83]
	MAPbI_3_/ Si	780 nm	0.013 mW/cm^2^	0	12 ms (6.37 mW/cm^2^)	54 ms (6.37 mW/cm^2^)	3.5 × 10^7^	3.8 mA/W	[85]
	α-FAPbI_3_/MAPbI_3_	780 nm	26.63 μW/cm^2^	0	/	/	9.03 × 10^8^	28.60 μA/W	[86]
	4H-SiC Schottky junction	325 nm	23.44 mW/cm^2^	0	0.27 s	/	/	9.12 nA/mW	[87]
	Si/water	400 nm	6 μW/cm^2^	−2	20 ms	60 ms	2.18 × 10^14^	12.3 A/W	[88]
		400 nm	6 μW/cm^2^	0	20 ms	800 ms	1.33 × 10^13^	754 mA/W	
	fibrous phosphorus micropillar arrays	405 nm	0.018 mW/cm^2^	0	12 ms	14 ms	3.50 × 10^9^	151.03 mA/W	[90]
Pyro for light energy harvesting	Si/ZnO NW	1064 nm	4.8 mW/cm^2^	0	15 μs	21 μs	/	4.3 × 10^−4^ A/W	[91]
	ITO/ZnO NWs/P3HT/PEDOT:PSS/Ag	Output current and voltage enhanced by 18 and 152% by the pyro-phototronic effect.							[92]
	CIGS/CdS/ZnO	Energy conversion efficiency first enhanced from 13.48 to 14.23% by the pyro-phototronic effect and further enhanced from 14.23 to 14.37% via the piezo-phototronic effect.							[93]
Pyro in Ferro materials for light energy harvesting	BaTiO_3_	405 nm	25.4 mW/cm^2^	0	6.9 s	24.5 s	3 × 10^5^	3 × 10^−7^ A/W	[94]
	Ag/BTO/Ag	365 nm	85.4 mW/cm^2^	0	0.5 s	23 s	9.8 × 10^4^	6.1 × 10^−8^ A/W	[95]
	ITO/BTO/Ag	405 nm	7.78 mW/cm^2^	0	0.88 s (127.6 mW/cm^2^)	1.06 s (127.6 mW/cm^2^)	8 × 10^9^	7.5 × 10^−6^ A/W	[102]
	ITO/BiFeO_3_/Ag	450 nm	0.86 mW/cm^2^	0	0.5 s (65.0 mW/cm^2^)	0.8 s (65.0 mW/cm^2^)	8.5 × 10^8^	2.5 × 10^−7^ A/W	[105]
	Ag/PZT/Ag	365 nm	140.1 mW/cm^2^	0	2.6 s	22.6 s	/	1.05 V/W	[107]
	hybrid quasi-freestanding structure of organic ferroelectric poly(vinylidene fluoride–trifluoroethylene) (P(VDF-TrFE)) with MoS_2_	637 nm	100 nW	5	480 μs	320 μs	9 × 10^14^	3260 A/W	[108]
	Ag NWs/HfO_2_/SiO_2_/Si	940 nm	4 mW/cm^2^	0	/	60 μs	/	68 μA/W	[109]
	Ag/Bi/2D PMA_2_PbCl_4_ MMB/Bi/Ag	320 nm	31.8 μW/cm^2^	0	162 μs	226 μs	1.01 × 10^11^	9 A/W	[110]
	Ag/Bi/(PMA)_2_PbCl_4_ microbelt arrays/Bi/Ag	320 nm	1.59 μW/cm^2^	0	73 μs (159.2 μW/cm^2^)	52 μs (159.2 μW/cm^2^)	2.3 × 10^6^	155.5 mA/W (0.71% strained)	[111]
Coupling of piezo- and pyro-phototronic	PEDOT:PSS/ZnO	325 nm	2.30 mW/cm^2^	0	/	/	7.5 × 10^9^ (−0.45% strained)	3.5 mA/W (−0.45% strained)	[43]
	ZnO/CuO NRs with Au NPs	325 nm	140 nW/cm^2^	0	18 ms (64.7 N)	12 ms (64.7 N)	3.3 × 10^13^ (73.7 N)	0.81 mA/W (73.7 N)	[50]
	Glass/Mo/Cu(In,Ga)Se_2_/CdS/ ZnO NW/ITO	808 nm	1.27 mW/cm^2^	0	50.17 us	60.65 us	1.17 × 10^13^ (pyro + piezo)	7.22 A/W (pyro + piezo)	[51]
Coupling of plasmonic and pyro-phototronic	ITO/Au NPs/ZnO/Au	365 nm	0.5 mW/cm^2^	−4	73 μs (0 V)	15 μs (0 V)	8.18 × 10^11^	4.68 A/W	[71]
	ITO/TiN NPs/ZnO	365 nm	1 mW/cm^2^	0	21 μs	27 μs	/	50 mA/W	[73]
	Au NP enriched GO/ZnO	365 nm	2 mW	0.006	29.1 μs	/	/	6.53 mA/W @ 1.5 V	[72]
	Si/Zn_1−x_Mn_x_O	900 nm	24 μW/cm^2^	0	3.4 ms	4.1 ms	4 × 10^13^	140 mA/W	[74]
	plasma-polymerized aniline–crystalline rubrene (PPA-CRB) thin film	450 nm	1 mW/cm^2^	0.5	4.8 ms	16.4 ms	/	/	[80]
	Au NPs@PPA-CRB	365 nm	/	1	10 ms (0.05 V, 405 nm)	7 ms (0.05 V, 405 nm)	/	(1.1 ± 0.036) mA/W	[81]

## Data Availability

Not applicable.
